# A single-dose live attenuated chimeric vaccine candidate against Zika virus

**DOI:** 10.1038/s41541-021-00282-y

**Published:** 2021-01-29

**Authors:** Wei-Xin Chin, Regina Ching Hua Lee, Parveen Kaur, Tian Sheng Lew, Thinesshwary Yogarajah, Hao Yuin Kong, Zi-Yun Teo, Cyrill Kafi Salim, Rong-Rong Zhang, Xiao-Feng Li, Sylvie Alonso, Cheng-Feng Qin, Justin Jang Hann Chu

**Affiliations:** 1 Laboratory of Molecular RNA Virology and Antiviral Strategies, Department of Microbiology and Immunology, Infectious Diseases Translational Research Programme, Yong Loo Lin School of Medicine, National University Health System, National University of Singapore, MD4 Level 5, 5 Science Drive 2, Singapore, 117597 Singapore; 2 NUSMed Biosafety Level 3 Core Facility, Yong Loo Lin School of Medicine, National University of Singapore, 14 Medical Drive, Singapore, 117599 Singapore; 3 Department of Virology, State Key Laboratory of Pathogen and Biosecurity, Beijing Institute of Microbiology and Epidemiology, Beijing, 100071 China; 4 Infectious Diseases Translational Research Programme, Department of Microbiology and Immunology, Yong Loo Lin School of Medicine, Singapore, Singapore; 5 Immunology program, Life Sciences Institute, National University of Singapore, Singapore, Singapore; 6 Institute of Molecular and Cell Biology, Agency for Science, Technology and Research (A*STAR), 61 Biopolis Drive, Proteos #06-05, Singapore, 138673 Singapore

**Keywords:** Dengue virus, Live attenuated vaccines, DNA vaccines

## Abstract

The mosquito-borne Zika virus is an emerging pathogen from the *Flavivirus* genus for which there are no approved antivirals or vaccines. Using the clinically validated PDK-53 dengue virus vaccine strain as a backbone, we created a chimeric dengue/Zika virus, VacDZ, as a live attenuated vaccine candidate against Zika virus. VacDZ demonstrates key markers of attenuation: small plaque phenotype, temperature sensitivity, attenuation of neurovirulence in suckling mice, and attenuation of pathogenicity in interferon deficient adult AG129 mice. VacDZ may be administered as a traditional live virus vaccine, or as a DNA-launched vaccine that produces live VacDZ in vivo after delivery. Both vaccine formulations induce a protective immune response against Zika virus in AG129 mice, which includes neutralising antibodies and a strong Th1 response. This study demonstrates that VacDZ is a safe and effective vaccine candidate against Zika virus.

## Introduction

Zika virus (ZIKV) is an emerging mosquito-borne pathogen from the genus *Flavivirus*^1–3^, which also contains other arthropod-borne pathogens such as yellow fever virus (YFV), dengue virus type 1 to 4 (DENV1 to DENV4), and Japanese encephalitis virus (JEV)^4^. ZIKV is typically spread by the *Aedes aegypti* and *Aedes albopictus* mosquitoes, although past outbreaks have also been linked to the *Aedes hensilli* mosquito^5,6^. ZIKV recently emerged to cause a larger outbreak in the Americas^1–3^. Between January 2015 and May 2017, ZIKV is estimated to have caused an estimated 8.5 million symptomatic infections in Brazil alone^3^.

ZIKV infection is reported to be asymptomatic in a majority of patients, with the reported asymptomatic rate varying from 29% to 82%, and with 80% being the commonly cited figure^7^. Symptomatic ZIKV infection typically presents as an acute and self-limiting febrile disease, with symptoms such as maculopapular rash, joint pain, and conjunctivitis^1,2^. In some patients, viable ZIKV can be recovered from patients weeks after the acute phase of the infection, and during this persistent infection phase ZIKV remains transmissible through sexual contact^8^. ZIKV infection is also associated with other severe complications. Infection in adults is associated with Guillain-Barré syndrome, and infection of pregnant women during the first two trimesters is associated with congenital microcephaly in newborns^1–3^. There are currently no approved vaccines or antiviral drugs for the prevention or treatment of ZIKV infection.

Flaviviruses share a common genome organisation, consisting of a positive sense single-stranded RNA genome that encodes for a single ORF that is flanked by 5′ and 3′ untranslated regions (5′UTR and 3′UTR)^4^. The ORF encodes for a polyprotein that is processed by host and viral proteases into the capsid, premembrane and envelope structural proteins (cap, prM and Env), as well as 7 non-structural proteins (NS1, NS2A, NS2B, NS3, NS4A, NS4B, and NS5)^4^. The flavivirus structural proteins are functionally interchangeable to a certain extent, and it is possible to construct viable chimeric viruses which express the heterologous structural proteins of another flavivirus^9–11^.

There are successful live attenuated vaccines against YFV and JEV^12^. In particular, the live attenuated yellow fever vaccine that has been in use since 1938 is considered a gold standard as a safe and effective antiviral vaccine^12^. Live attenuated vaccines against DENV have also demonstrated good attenuation during clinical trials^13–15^. Live attenuated vaccines are highly immunogenic compared to inactivated vaccines^12^. For example the live JEV vaccine that is based on the SA14-14-2 strain only requires one dose, whereas inactivated JEV vaccines require multiple doses^12^. Therefore, a live attenuated vaccine against ZIKV would be a promising strategy for controlling ZIKV.

However, one major concern is that live vaccines that look promising during initial preclinical studies may show inadequate attenuation once they reach human clinical trials^16^. One way of addressing this risk is to repurpose a clinically validated vaccine strain to serve as a viral backbone for a chimeric virus^9–11,13–15,17,18^. Such a chimera would express the attenuated replication machinery of the vaccine strain, whilst also expressing the major structural antigens of the target virus. This strategy has been used in the development of several live attenuated tetravalent dengue vaccines, with the Sanofi-Pasteur, Takeda, and NIH dengue vaccines all containing at least one chimeric virus^13–15,17,18^. In particular, the Takeda TAK-003 (or DENVax) dengue vaccine that has recently completed phase II and III clinical trials contains four component viruses, one of which is the DENV2-PDK-53 vaccine strain that was developed by Mahidol University, Thailand^9,10,13,14,18,19^. The other three component viruses of TAK-003 are chimeric viruses that utilise DENV2-PDK-53 as their viral backbone.

Several studies have adopted the YFV-17D vaccine strain to serve as the backbone of a chimeric virus vaccine against ZIKV^20–22^, while another study adopted the JEV-SA14-14-2 vaccine strain for the same reason^23^. However, the mutations with the largest impact on attenuating the neurovirulence of JEV-SA14-14-2 lie within the Env protein, and these attenuating mutations are lost during chimerisation^24^. The situation is even more uncertain with the YFV-17D, because its attenuating mutations have yet to be identified^25^. Furthermore, live viral vaccines face several disadvantages in terms of genetic stability, storage, and scalability of production during sudden outbreaks^26–28^. These shortcomings resulted in a shortage of the live yellow fever vaccine during the 2016 YFV outbreak in Angola as the short shelf life of the vaccine had limited the size of stockpiles and manufacturers were unable to scale up production capacity in time^28^.

**Fig. 1:**
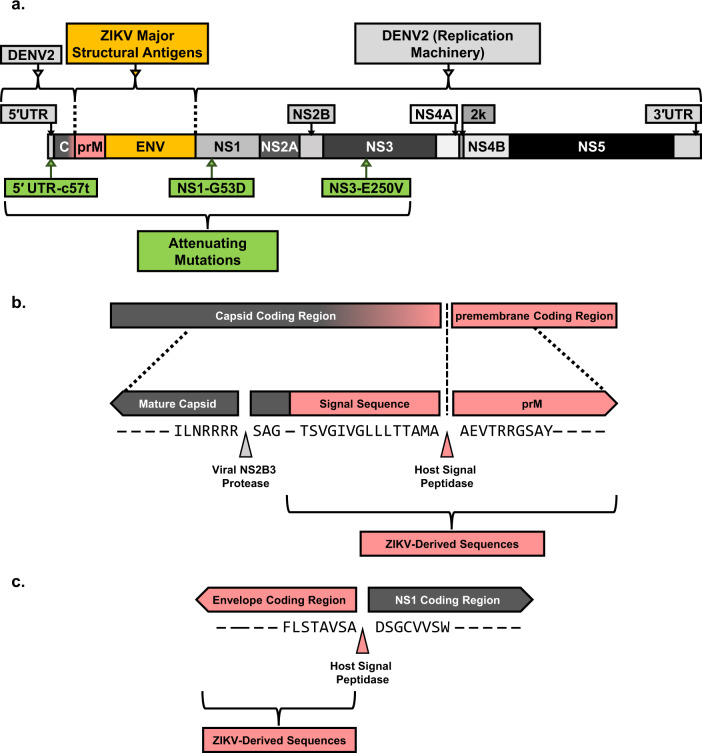
Genomic map and signal sequences of chimeric Zika/dengue virus VacDZ. **a** Genomic map of VacDZ. 5′ & 3′UTR: untranslated regions. Protein coding regions: C, capsid protein; prM, premembrane protein; Env, envelope protein; NS1 to NS5, non-structural proteins 1 to 5. ZIKV-PRVABC59 derived sequences are shown in red and gold. **b** Premembrane protein signal sequence at the capsid-premembrane junction. The C-terminus of the capsid protein contains the premembrane signal sequence, and in VacDZ this signal sequence is ZIKV derived. Grey and red triangles indicate cleavage sites of viral NS2B3 protease and host signal peptidase respectively. **c** Signal sequence at the junction of the Envelope and NS1 proteins. Red triangle indicates cleavage site of host signal peptidase.

To address these problems, we developed a chimeric dengue/Zika virus called VacDZ as a vaccine against ZIKV (Fig. 1). DENV2-PDK-53 vaccine strain was chosen as a viral vaccine backbone because the attenuating mutations of DENV2-PDK-53 are located outside the prM and Env protein coding regions, meaning they are not affected by chimerisation^19,29,30^. The attenuating mechanism for one of these key attenuating mutations, NS1-G53D, was recently determined to be the enhanced induction of the type-I interferon response by the mutant NS1-G53D protein, a mechanism that should not be affected by chimerisation^31^. Furthermore, clinical trials have shown that chimeric vaccines that are derived from DENV2-PDK-53 have excellent safety profiles^13,14^. All this indicates that VacDZ is highly likely to retain the attenuation of the DENV2-PDK-53 backbone. We also developed VacDZ so that it can be delivered as either a live virus, or as a DNA-launched vaccine that produces live, functional VacDZ in vivo^32–34^. Here, we report the development of VacDZ as a safe and effective vaccine against ZIKV.

## Results

### Construction of infectious clone of VacDZ chimeric dengue/Zika virus

We constructed the infectious clone of our chimeric dengue/Zika virus, VacDZ, (Fig. 1), by modifying our existing DENV2-16681 infectious clone^35^. The infectious clone of VacDZ, called pVacDZ, was constructed using conventional molecular cloning techniques (Supplementary Figure 1).

The 16681 strain of DENV2 is the parent strain of PDK-53. While PDK-53 differs from the parental wildtype DENV2-16681 by several mutations, only three mutations are necessary and sufficient for the attenuation of PDK-53: 5′UTR-c57t, NS1-G53D, and NS3-E250V^9–11,29^. Therefore, we only introduced these three attenuating mutations into our infectious clone to construct pVacDZ (Fig. 1, Supplementary Figure 1). We also replaced the region encoding for the prM signal sequence, prM protein, and Env protein with their counterpart from ZIKV strain PRVABC59 (Fig. 1b). The prM signal sequence at the capsid-prM junction was replaced because prior studies with dengue/West Nile virus chimeras showed that the prM protein requires a homologous signal sequence upstream for correct processing^11^. The signal sequence at the Env-NS1 junction is also shown (Fig. 1c).

Flaviviral infectious clone plasmids can be highly unstable, and this instability can prevent the construction of infectious clones for chimeric vaccines^10,19^. Therefore, we adopted two strategies in order to stabilise our pVacDZ infectious clone. First, we utilised an inducible CMV promoter (TRE-minCMV) that has been shown to stabilise flavivirus infectious clones^35,36^. Second, we cloned an intron sequence into the NS1 coding region^37^.

We also constructed a ΔGDD mutant of VacDZ as a control for DNA vaccination studies (Supplementary Figure 2). VacDZ-ΔGDD has a lethal deletion of the ‘glycine-aspartic acid-aspartic acid’ catalytic triad of the NS5 RNA-dependent RNA polymerase (RDRP)^4^. While VacDZ-ΔGDD can express a small quantity of viral proteins, it cannot enter into the exponential RNA-replication and protein expression stage of the virus replication cycle.

### Rescue of VacDZ by DNA-launch

Virus rescue for our infectious clones takes place by DNA-launch, whereby the infectious clone plasmid is co-transfected with a pTet-Off Advanced accessory plasmid^35,36^. After transfection, the accessory plasmid activates the TRE-minCMV promoter of the infectious clone, which then recruits host nuclear machinery to transcribe the viral RNA. The viral RNA is eventually processed into a functional viral genome that can establish a normal virus replication cycle. VacDZ was rescued by DNA-launch in BHK-21 cells to produce a seed stock. VacDZ seed stock was then amplified by passaging the virus in BHK-21 cells to produce a working stock, which was then titrated by plaque assay.

### VacDZ expresses ZIKV envelope protein

We first verified if VacDZ was expressing the ZIKV Env protein. BHK-21 cells were infected with VacDZ or with parental DENV2-16681 or ZIKV. Mock infected cells were used as a control. The cells were then fixed and viral protein expression was analysed using immunofluorescence assay. Cells that were infected with VacDZ, DENV2-16681, or ZIKV were found to express flaviviral NS1 protein, whereas mock infected cells did not (Supplementary Figure 3). Cells that were infected with VacDZ or ZIKV were also found to express ZIKV Env protein, while mock infected cells and DENV2-16681 infected cells did not (Supplementary Figure 3). This demonstrates that VacDZ expresses ZIKV Env protein as intended.

### VacDZ retains the small plaque phenotype of PDK-53

In order to validate the potential safety of VacDZ, we investigated if VacDZ retains the key markers of attenuation of the DENV2-PDK-53 vaccine strain, namely small plaque phenotype, temperature sensitivity, attenuation of neurovirulence in suckling mice, and attenuation of pathogenicity in AG129 mice^9–11,19,29^.

**Fig. 2:**
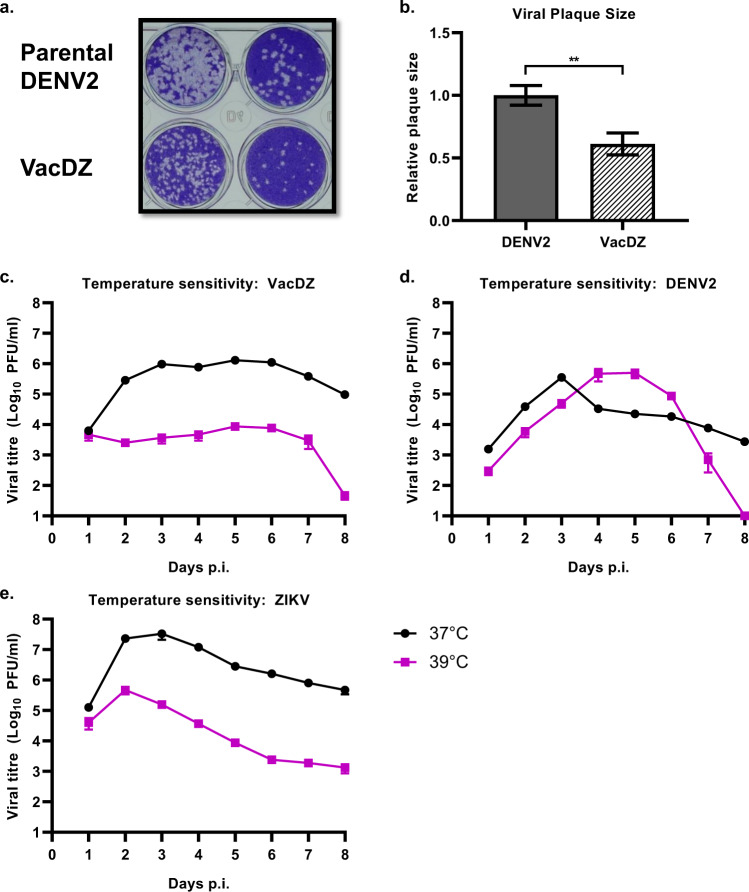
Plaque formation and temperature sensitivity assay. VacDZ was investigated for markers of attenuation in cell culture. **a** Plaque formation assay in BHK-21 cells for VacDZ and parental DENV2-16681 to investigate small plaque phenotype. Incubation time for both viruses was six days. Plaques were then visualized by crystal violet staining. **b** Comparison of mean plaque sizes for VacDZ and parental DENV2-16681, measured by pixel width. Data represents mean of 10 plaques. Statistical analysis was performed using one way ANOVA. Statistical significance: *, *P* < 0.05; **, *P* < 0.01; ***, *P* < 0.001. Temperature sensitivity assay for **c** VacDZ, **d** DENV2-16681, and **e** ZIKV was performed by comparing the viral replication kinetics in BHK-21 cells at 37 °C and 39 °C (black and magenta lines respectively). Viral replication titres were analysed using plaque assay. Temperature sensitivity is defined as a reduction in viral titre (PFU/ml) of 1-log or more at 39 °C relative to 37 °C. Data represents the mean of three replicates.

We started by investigating if VacDZ retains the small plaque phenotype. We compared the plaque formation phenotype of VacDZ and parental wildtype DENV2-16681 by performing a plaque assay in BHK-21 cells. VacDZ formed significantly smaller plaques than DENV2-16681 (Figs. 2a, b), confirming that VacDZ retains the small plaque phenotype.

### VacDZ replication kinetics and temperature sensitivity

Next, we investigated the replication kinetics of VacDZ in comparison to the parental wildtype DENV2-16681 and ZIKV. We also investigated if VacDZ retains the temperature sensitivity phenotype, which is an attenuation marker that is defined as a reduction of the viral titre of 1-log or more at 39 °C relative to 37 °C^11^.

We compared the growth kinetics of these viruses in four cell lines which are commonly used for flavivirus studies: BHK-21 baby hamster kidney cells, Vero African green monkey kidney cells, Huh-7 human hepatoma cells, and C6/36 *Aedes aegypti* mosquito cells. The cells were infected with VacDZ, DENV2-16681, or ZIKV. After infection, the supernatant was harvested daily, and the extracellular virus titre was determined by using plaque assay. Temperature sensitivity was investigated in BHK-21 cells.

In BHK-21 cells, ZIKV had the highest peak titre, followed by VacDZ and then DENV2-16681 (3.33 × 10^7^, 1.30 × 10^6^, and 3.53 ×10^5^ PFU/ml respectively) (Figs. 2c-e). Both VacDZ and ZIKV were temperature sensitive, whereas DENV2-16681 was not (Fig. 2c-e).

In Vero cells, DENV2-16681 had the highest peak titre, followed by ZIKV and then VacDZ (2.00 × 10^6^, 1.93 × 10^6^, and 1.90 × 10^6^ PFU/ml respectively) (Supplementary Figure 4a). In Huh-7 cells, ZIKV had the highest peak titre, followed by VacDZ and then DENV2-16681 (4.33 × 10^6^, 1.13 × 10^6^, and 4.63 × 10^5^ PFU/ml respectively) (Supplementary Figure 4b). For viral replication in mammalian cells, VacDZ generally seems to replicate to titres which are intermediate to the parental DENV2-16681 and ZIKV. In C6/36 mosquito cells, ZIKV had the highest peak titre, followed by DENV2-16681 and then VacDZ (7.33 × 10^7^, 2.30 × 10^7^, and 2.53 × 10^5^ PFU/ml respectively) (Supplementary Figure 4c). This demonstrates that VacDZ has attenuated replication kinetics in mosquito cells, which is a predictive marker for reduced transmissibility in mosquitoes^11^. This is ideal since vaccine strains should not be transmissible by mosquitoes^11^.

### VacDZ is genetically stable

The potential safety of VacDZ is dependent on its three attenuating mutations: 5′UTR-c57t, NS1-G53D and NS3-E250V. These attenuating mutations are known to be stable for the component viruses of the TAK-003 live dengue vaccine, which also utilise the DENV2-PDK-53 backbone^19,30^. Therefore, we investigated if these attenuating mutations were also stable in VacDZ. We serially passaged VacDZ in BHK-21 cells. At passage 10, we extracted the viral RNA and analysed the reversion rates of the attenuating mutations using next-generation sequencing (Omics Drive Pte Ltd). All three attenuating mutations had reversion rates of less than 1% (Supplementary Table 1). This degree of genetic stability is similar to DENV2-PDK-53 and other DENV2-PDK-53 derived chimeras^19^. This demonstrates that VacDZ is genetically stable, with a low risk of reversion.

### VacDZ retains attenuation of neurovirulence in suckling mice

We proceeded to evaluate the in vivo safety and efficacy of VacDZ using two different mouse models. We used a suckling mouse model to evaluate the attenuation of neurovirulence, while the interferon deficient AG129 mouse model was used to evaluate the overall attenuation and immunogenicity of VacDZ^9–11,19^.

**Fig. 3:**
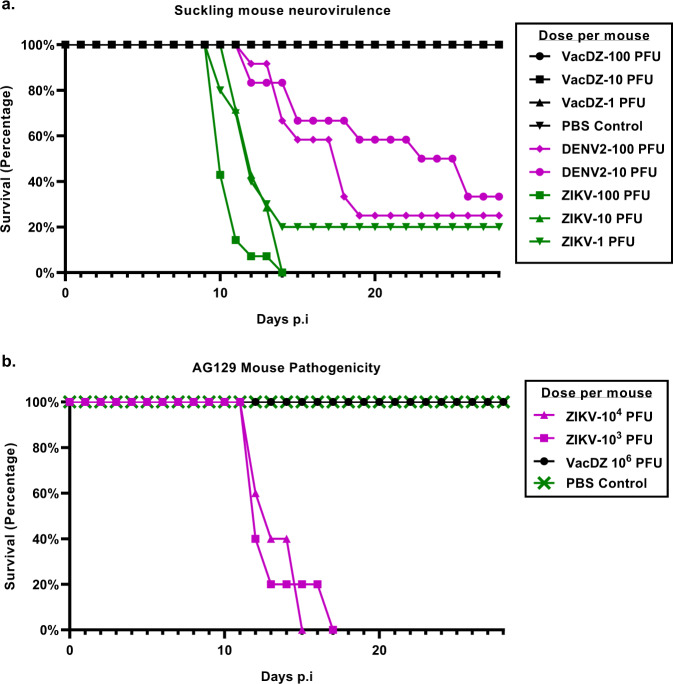
Survival curve for neurovirulence and pathogenicity studies in mice. **a** Neurovirulence studies in suckling mice. Newborn outbred white ICR mice were challenged with different doses of VacDZ, DENV2-16681, or ZIKV via intracranial inoculation. The mice were kept for four weeks and observed daily for clinical symptoms and killed when they reached a humane endpoint. Group sizes: PBS control, *n* = 6; VacDZ, *n* = 11 for all titres; DENV2, *n* = 12 for all titres; ZIKV-100 PFU, *n* = 14; ZIKV-10 PFU, *n* = 7; ZIKV-1 PFU, *n* = 10. **b** Pathogenicity studies in adult AG129 mice. 5-8 weeks old AG129 mice were challenged with the indicated doses of VacDZ or ZIKV or PBS control via intraperitoneal inoculation. Doses indicated are per mouse. Group sizes were *n* = 5 for ZIKV challenge, and *n* = 6 for VacDZ or PBS challenge. The mice were kept for four weeks and observed daily for clinical symptoms and killed when they reached a humane endpoint.

Newborn outbred white ICR mice were challenged by intracranial inoculation within 24 h of birth with different doses of VacDZ, DENV2-16681, ZIKV, or a PBS vehicle control. The mice were then observed daily for clinical signs for a period of four weeks (Fig. 3a, Supplementary Table 2).

The clinical signs that were commonly observed in mice that were challenged with ZIKV or DENV2-16681 were weight loss, wobbling gait, limb weakness or dragging, paralysis, hunching, or inactivity. Mice that were challenged with 1, 10, or 100 PFU of ZIKV per mouse had a mortality rate of 80%, 100%, or 100% respectively. Mice that were challenged with 10 or 100 PFU of DENV2-16681 per mouse had a mortality rate of 66.7% or 75% respectively. In contrast, mice that were challenged with 1, 10, or 100 PFU of VacDZ per mouse or with the PBS vehicle control had a mortality rate of 0%. This demonstrates that VacDZ has highly attenuated neurovirulence in suckling mice compared to the parental wildtype DENV2-16681 and ZIKV.

### VacDZ has attenuated pathogenicity in AG129 mice

We proceeded to evaluate VacDZ for safety and efficacy in the interferon deficient AG129 mouse model. AG129 mice are commonly used for flavivirus vaccine studies because they are highly susceptible to lethal flavivirus challenge, but retain the ability to develop an adaptive immune response^10,38,39^.

ZIKV strain PRVABC59 (ZIKV-PRVABC59) is known to be lethal for AG129 mice, whereas DENV2-16681 is not^38,40^. We started by investigating the pathogenicity of ZIKV-PRVABC59 in AG129 in order to determine a suitable dose for subsequent lethal challenge studies. Because VacDZ encodes the prM and Env proteins of ZIKV-PRVABC59, we also investigated if expression of these ZIKV proteins resulted in any gain of pathogenicity for VacDZ.

AG129 mice were challenged by intraperitoneal inoculation with different doses of VacDZ, ZIKV, or PBS vehicle control. The mice were then observed daily for clinical symptoms for a period of four weeks (Fig. 3b). Mice that were challenged with 10^3^ or 10^4^ PFU of ZIKV per mouse had a mortality rate of 100%. The most commonly observed clinical signs in mice that were challenged with ZIKV was weight loss, with most of the mice reaching a humane endpoint of cumulative 20% weight loss relative to the initial weight. In contrast, mice that were challenged with 10^6^ PFU of VacDZ per mouse or with the PBS vehicle control had a mortality rate of 0%. The changes in mouse weights are shown in Supplementary Figure 5. This demonstrates that VacDZ has attenuated pathogenicity in AG129 mice as well.

### VacDZ is immunogenic in AG129 mice

Next, we investigated if vaccination with VacDZ could induce an immune response against ZIKV in the form of neutralising antibodies or a T helper cell response.

We tested VacDZ as both a live virus vaccine (live VacDZ) and also as a DNA-launched vaccine (DNA-launched VacDZ). The DNA-launched vaccine is essentially an alternative delivery method, whereby the pVacDZ infectious clone is transfected in vivo, after which the production of live VacDZ occurs in vivo^32,33^. This live, DNA-launched VacDZ can then proceed to establish a virus replication cycle as per normal.

Most DNA-launched flavivirus vaccines reported to date have used electroporators for in vivo transfection^32,33^. In order to simplify our DNA vaccination protocol, we used the in vivo-jetPEI transfection reagent (Polyplus transfection) since it does not require an electroporator for delivery.

For live VacDZ, the dose for vaccination or boosting was 10^4^ PFU of VacDZ per mouse, and PBS was used as a vehicle control. For DNA-launched VacDZ, the dose for vaccination or boosting was 80 μg of pVacDZ and 20 μg of pTet-Off Advanced per mouse, while a dose of 80 μg of pVacDZ-ΔGDD mutant and 20 μg of pTet-Off Advanced per mouse was used for the RDRP-defective control, and a 5% glucose solution was used as a vehicle control.

AG129 mice were first vaccinated with live VacDZ, DNA-launched VacDZ, or their respective controls via intraperitoneal inoculation. Four weeks after primary vaccination, the mice were boosted with the same dose of their respective vaccine or control. Four weeks after boosting, the mice were killed and their blood and organs were harvested.

**Fig. 4:**
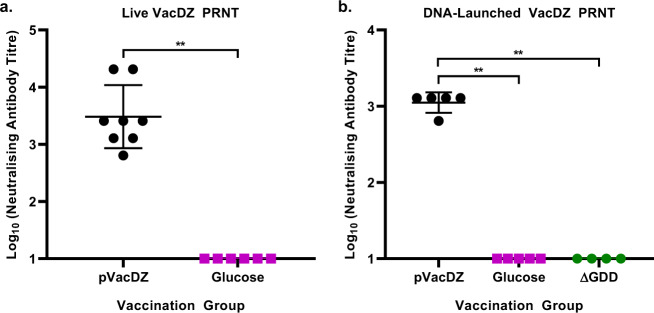
ZIKV neutralising antibody titres in vaccinated AG129 mice. 5-8 weeks old AG129 mice were inoculated intraperitoneally with the indicated vaccine or control and then boosted four weeks later with the same dose of their respective vaccine or control. Four weeks after boosting mouse serum was harvested. ZIKV neutralising antibody titres were determined using PRNT, and is defined as the highest serum dilution that reduced the ZIKV plaque count by at least 50% in three technical replicates. Each point represents the neutralising antibody titre for one mouse. **a** PRNT for AG129 mice vaccinated with live VacDZ (10,000 PFU per mouse, *n* = 8) or PBS vehicle control (*n* = 6). **b** PRNT for AG129 mice vaccinated with DNA-launched VacDZ (pVacDZ, 80 μg of pVacDZ and 20 μg of pTet-Off Advanced per mouse, *n* = 5), 5% glucose solution vehicle control (glucose, *n* = 5), or pVacDZ-ΔGDD replication defective mutant (ΔGDD, 80 μg of pVacDZ-ΔGDD and 20 μg of pTet-Off Advanced per mouse, *n* = 4). Statistical analysis was performed using one way ANOVA, and post-hoc analysis was performed using Tukey HSD. Statistical significance: *, *P* < 0.05; **, *P* < 0.01; ***, *P* < 0.001.

To investigate if there was induction of neutralising antibodies in the vaccinated mice, we analysed their serum using plaque reduction neutralisation test (PRNT). AG129 mice that were vaccinated with live VacDZ or with DNA-launched VacDZ were all found to have neutralising antibodies against ZIKV (100% seroconversion rate), with live VacDZ inducing a stronger response, though this difference was not significant (Fig. 4, Supplementary Table 3). In contrast, AG129 mice vaccinated with the PBS vehicle control, 5% glucose solution vehicle control, or pVacDZ-ΔGDD mutant control all had a 0% seroconversion rate (Fig. 4, Supplementary Table 3).

Live VacDZ vaccinated mice had anti-ZIKV PRNT titres ranging from 1:640 to 1:20480 (geometric mean titre of 3044.37) (Fig. 4a). DNA-launched VacDZ vaccinated mice had anti-ZIKV PRNT titres ranging from 1:640 to 1:1280 (geometric mean titre of 1114.3) (Fig. 4b).

**Fig. 5:**
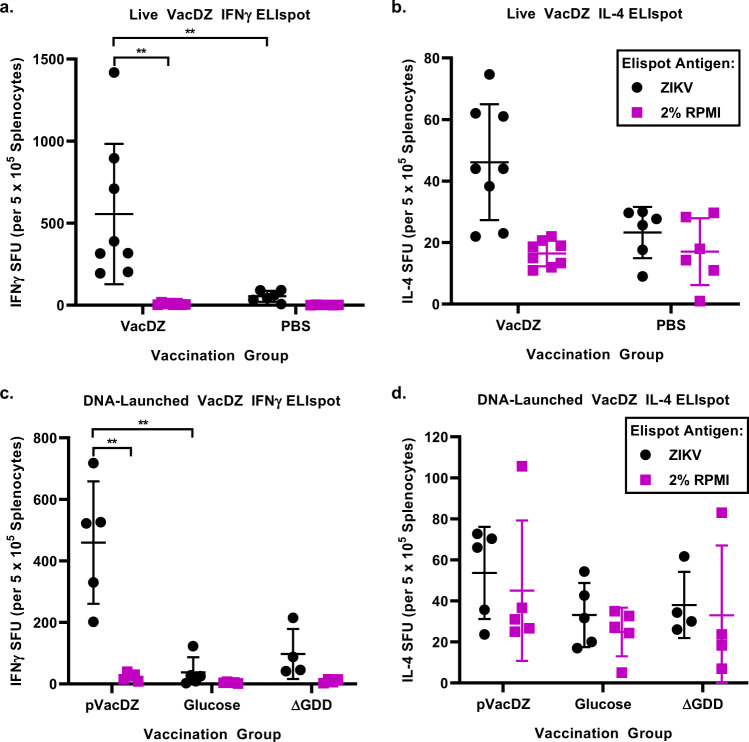
ELIspot assay of T helper cell response. 5-8 weeks old AG129 mice were inoculated intraperitoneally with the indicated vaccine or control and then boosted four weeks later with the same dose of their respective vaccine or control. Four weeks after boosting the mouse splenocytes were harvested. IFNγ-ELIspot was used to analyse the Th1 response, and IL-4-ELIspot was used to analyse the Th2 response. Each point represents one mouse and is the mean spot forming unit (SFU) count of three technical replicates. Magenta circles: activated with ZIKV, black squares: activated with RPMI medium control. **a**, **b** IFNγ and IL-4 ELIspot assays respectively for AG129 mice vaccinated with live VacDZ (10,000 PFU per mouse, *n* = 8) or PBS vehicle control (*n* = 6). **c**, **d** IFNγ and IL-4 ELIspot assays respectively for AG129 mice vaccinated with DNA-launched VacDZ (pVacDZ, 80 μg of pVacDZ and 20 μg of pTet-Off Advanced per mouse, *n* = 5), 5% glucose solution vehicle control (glucose, *n* = 5), or pVacDZ-ΔGDD replication defective mutant (ΔGDD, 80 μg of pVacDZ-ΔGDD and 20 μg of pTet-Off Advanced per mouse, *n* = 4). Statistical analysis was performed using one way ANOVA, and post-hoc analysis was performed using Tukey HSD. Statistical significance: *, *P* < 0.05; **, *P* < 0.01; ***, *P* < 0.001.

To investigate if there was induction of a type 1 or type 2 T helper cell (Th1 or Th2) response in the vaccinated mice, we analysed their splenocytes by using IFNγ or IL-4 ELIspot assays respectively. The mouse splenocytes were activated using ZIKV at a MOI of 0.5, or with control RPMI medium supplemented with 2% FCS. AG129 mice that were vaccinated with live VacDZ developed a significant Th1 and Th2 response (Fig. 5a, b). However, the Th1 response was stronger compared to the Th2 response. For the mice vaccinated with live VacDZ, the Th1 response was 10.2-fold higher compared to the mice vaccinated with the PBS control, whereas the Th2 response was only 2-fold higher.

Mice that were vaccinated with DNA-launched VacDZ developed a significant Th1 response, but no significant Th2 response (Fig. 5c and d). As expected, mice that were vaccinated with the ΔGDD mutant control did not develop any significant Th1 or Th2 response compared to the 5% glucose solution vehicle control (Fig. 5c and d). For the mice vaccinated with DNA-launched VacDZ, the Th1 response was 12.2-fold higher compared to the mice vaccinated with the 5% glucose solution control.

This demonstrates that both live VacDZ and DNA-launched VacDZ induce an immune response against ZIKV in the form of neutralising antibodies and a Th1 response. This Th1 biased response and the associated IFNγ response are favourable because they are associated with protective immunity against flaviviruses, as well as milder disease outcomes during flavivirus infection^41–43^.

### VacDZ confers protective immunity against lethal ZIKV challenge

Finally, we investigated if vaccination with VacDZ confers protective immunity against lethal ZIKV challenge. For the protective immunity studies, we adopted a single dose regime that did not involve any boosting: mice were vaccinated with a single dose of vaccine or control, and then challenged with ZIKV.

AG129 mice were vaccinated with live VacDZ, DNA-launched VacDZ, or their respective controls, using the same dose described above for immunogenicity studies. Four weeks after vaccination the mice were challenged with a lethal dose of ZIKV (10^5^ PFU per mouse). The mice were then observed daily for clinical signs for a period of four weeks. Four weeks after lethal challenge, the mice were killed and their blood and organs were harvested.

**Fig. 6:**
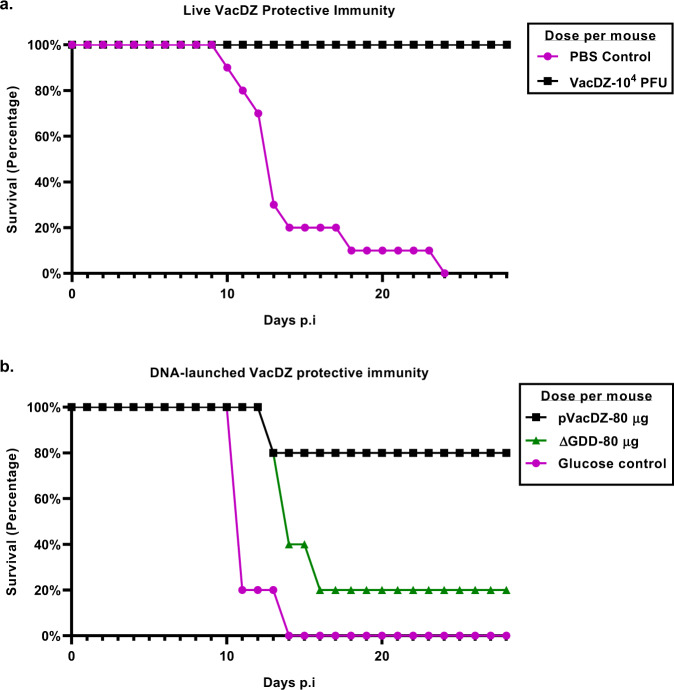
Survival curve for protective immunity studies in AG129 mice. 5-8 weeks old AG129 mice were inoculated intraperitoneally with the indicated vaccine or control. Four weeks after vaccination, they were challenged with a lethal dose of 10^5^ PFU of ZIKV per mouse. The mice were then kept for four weeks and observed daily for clinical symptoms and killed when they reached a humane endpoint. **a** AG129 mice vaccinated with VacDZ (10,000 PFU per mouse, *n* = 11) or PBS vehicle control (*n* = 10). **b** AG129 mice vaccinated with DNA-launched VacDZ (pVacDZ, 80 μg of pVacDZ and 20 μg of pTet-Off Advanced per mouse, *n* = 5), 5% glucose solution vehicle control (glucose, *n* = 5), or pVacDZ-ΔGDD replication defective mutant (ΔGDD, 80 μg of pVacDZ-ΔGDD and 20 µg of pTet-Off Advanced per mouse, *n* = 5).

Mice that were inoculated with the PBS or 5% glucose solution vehicle controls had a survival rate of 0% when challenged with ZIKV (Fig. 6, Supplementary Table 4). Mice that were vaccinated with the replication-defective pVacDZ-ΔGDD mutant control had a survival rate of 20% when challenged with ZIKV (Fig. 6b, Supplementary Table 4). In contrast, mice that were vaccinated with live VacDZ or DNA-launched VacDZ had a survival rate of 100% or 80% respectively when challenged with ZIKV (Fig. 6, Supplementary Table 4). Mouse weights are shown in Supplementary Figure 6. This demonstrates that both live and DNA-launched VacDZ confer protective immunity against ZIKV, with live VacDZ having better protective efficacy.

**Fig. 7:**
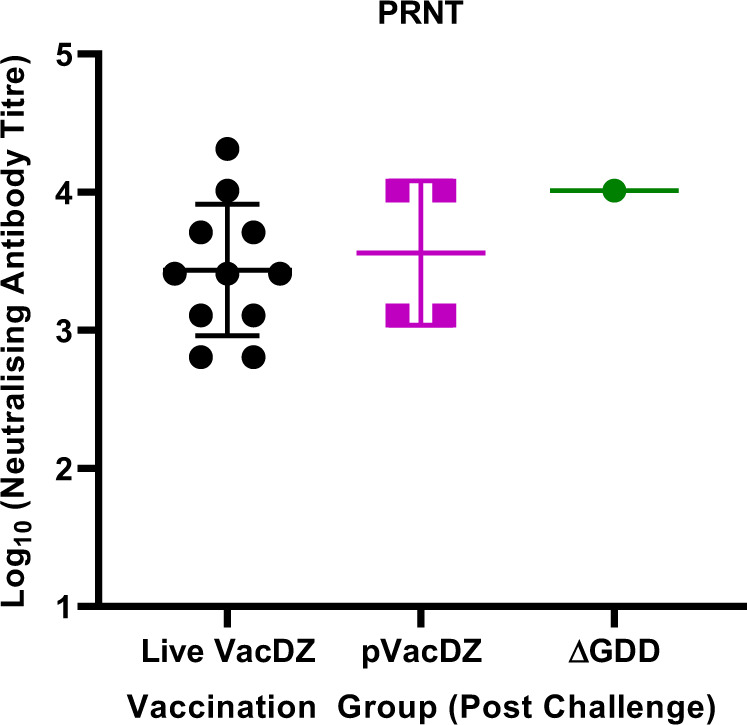
ZIKV neutralising antibody titres in vaccinated AG129 mice post challenge. 5-8 weeks old AG129 mice were inoculated intraperitoneally with a single dose of the indicated vaccines (or their respective control). Four weeks after vaccination, they were challenged with a lethal dose of 10^5^ PFU of ZIKV per mouse. The mice were kept for four weeks and observed daily for clinical symptoms and killed when they reached a humane endpoint. Four weeks after lethal challenge, serum was harvested from the surviving mice. ZIKV neutralising antibody titres were determined using PRNT, and is defined as the highest serum dilution that reduced the ZIKV plaque count by at least 50% in three technical replicates. Each point represents the neutralising antibody titre for one mouse. N numbers are for the number of mice surviving four weeks after lethal challenge. Live VacDZ dose was 10,000 PFU per mouse, n = 11. pVacDZ: DNA-launched VacDZ, dose was 80 μg of pVacDZ and 20 μg of pTet-Off Advanced per mouse, *n* = 4. ΔGDD: pVacDZ-ΔGDD replication defective mutant, dose was 80 μg of pVacDZ-ΔGDD and 20 μg of pTet-Off Advanced per mouse, *n* = 1.

Next, to investigate if there was induction of neutralising antibodies in the surviving mice, we analysed their serum by using PRNT. All surviving mice were found to have neutralising antibodies against ZIKV (seroconversion rate of 100% in surviving mice) (Fig. 7, Supplementary Table 4). Live VacDZ vaccinated mice had anti-ZIKV PRNT titres ranging from 1:640 to 1:20480 (geometric mean titre of 2726.51). Surviving DNA-launched VacDZ vaccinated mice had anti-ZIKV PRNT titres ranging from 1:1280 to 1:10240 (geometric mean titre of 3620.39).

Curiously, the pVacDZ-ΔGDD mutant that was used as the RDRP-defective negative control for DNA vaccination seemed to offer weak protection against ZIKV. For the immunogenicity studies in AG129 mice, the strongest Th1 response from the pVacDZ-ΔGDD vaccinated group was higher than the weakest Th1 response from the group vaccinated with DNA-launched VacDZ (mean spot forming counts of 215.0 and 201.7 respectively for IFNγ-ELIspot) (Fig. 5c). However, pVacDZ-ΔGDD vaccination did not induce any neutralising antibodies against ZIKV, even with a vaccination and boost regime (Fig. 4b). Therefore, we hypothesise that pVacDZ-ΔGDD is able to elicit a protective T-cell response in a minority of mice. This may have allowed one of the mice in the pVacDZ-ΔGDD vaccinated group to survive lethal challenge with ZIKV (Fig. 6b), and in the process the mouse was able to seroconvert as well (Fig. 7).

## Discussion

In this study, we developed a chimeric dengue/Zika virus called VacDZ as a vaccine candidate against ZIKV. VacDZ utilises the clinical validated DENV2-PDK-53 vaccine strain as the backbone of the chimeric virus^13,14^. This was to ensure that our vaccine would be sufficiently attenuated. Unlike other flavivirus vaccine strains, the attenuating mutations of DENV2-PDK-53 vaccine are known, and they are not affected by chimerisation of the structural proteins^29^. This is a similar approach to the chimeric rZIKV/D4Δ30-713 vaccine for ZIKV, which is derived from the live attenuated dengue 4 backbone, rDEN4Δ30^44^. At the time of writing, the rZIKV/D4Δ30-713 is set to undergo phase I clinical trials, but the preclinical data of the vaccine has not been published^44^. The attenuating mutations of both the DENV2-PDK-53 and rDEN4Δ30vaccine strains are known, and in each vaccine strain they are located outside the structural protein coding region^29,45^. Both DENV2-PDK-53 and rDEN4Δ30 have been successfully repurposed as backbones for chimeric vaccines^45–47^. The Takeda TAK-003 (or DENVax) tetravalent dengue vaccine contains DENV2-PDK-53 itself, as well as three chimeric viruses which utilise DENV2-PDK-53 as their backbone^13,14,18^. The NIH TV003 tetravalent dengue vaccine contains DEN4Δ30 itself, as well as a chimeric DENV2/4 which utilises rDEN4Δ30as its backbone^45–47^. However, the DENV2-PDK-53 vaccine strain is the more established vaccine backbone, as the Takeda TAK-003 has already completed phase II & III clinical trials, while the NIH TV003 vaccine has only completed phase II clinical trials^13,14,45–47^.

We found that VacDZ retained the key attenuation markers of the DENV2-PDK-53 vaccine strain: small plaque phenotype, temperature sensitivity, attenuation of neurovirulence in suckling mice, and attenuation of pathogenicity in AG129 mice. We found that both live VacDZ and DNA-launched VacDZ could induce a protective immune response against ZIKV. This immune response included neutralising antibodies against ZIKV, a strong Th1 (and IFNγ) response against ZIKV, and a weaker or negligible Th2 T-cell response. This Th1 biased response and the associated IFNγ response are favourable because they are associated with milder disease outcomes during flavivirus infections and with a protective CD8 + T-cell response^41–43^.

Because VacDZ is a chimeric vaccine that is derived from the DENV2-PDK-53 vaccine strain, we based our vaccine design and development process on prior studies which describe the development on other chimeric vaccines which are derived from DENV2-PDK-53^10,11,18^. Therefore, we adopted the interferon deficient AG129 mouse strain that was described in some of these prior studies because it serves as a highly reproducible lethal mouse model^10,18,39^. In particular, we found that live VacDZ and DNA-launched VacDZ were able to induce comparable neutralising antibody titres against their respective target viruses in AG129 mice (1:1114.3 to 1:3044.37) when compared to chimeric DENV2/1, DENV2/3, and DENV2/4 vaccines (1:320 to 1:2560)^10^. However, while the AG129 strain is a highly reproducible mouse model that is used in many flavivirus vaccine studies, the defective interferon response may affect its relevance as a model for pathogenic flavivirus infections in humans^10,38,39,48^. Therefore, in future studies we hope to evaluate our VacDZ vaccine in a non-human primate model or in a immunocompetent ZIKV mouse model, such as a recently developed human STAT2 knock-in mouse model^49,50^.

The World Health Organisation has published a target product profile (TPP) for ZIKV vaccines^51^. Two potential vaccination strategies are proposed. The first is a routine vaccination programme where there will be predictable demand, production, and delivery of the vaccine. In such circumstances, live VacDZ would be an ideal candidate. Live VacDZ had better efficacy compared to DNA-launched VacDZ. Furthermore, live attenuated virus vaccines, such as the YFV-17D vaccine, are a proven technology with a long, safe, and successful track record. The longer production time for live vaccines should not pose a problem given the predictable turnover of vaccine stocks. However, the WHO TPP is oriented towards the deployment of ZIKV vaccines in response to an outbreak. In these circumstances, DNA-launched VacDZ is not without its merits. DNA-launched VacDZ combines the advantages of DNA vaccines and live vaccines. First, DNA plasmids are more stable and easier to store, with simplified cold chain requirements^26–28,34^. While live VacDZ needs to be stored at -80 °C, DNA-launched VacDZ can be stored at -20 °C for many years, or even at 4 °C for a few months. This simplified storage requirement is one of the desired characteristics described in the WHO TPP for ZIKV vaccines^51^. Second, compared to live viral vaccines, it is easier to scale up the production of DNA vaccines during an outbreak^26–28^. Third, DNA-launched VacDZ is far more immunogenic compared to traditional DNA vaccines that express non-replicating antigens^26,27,34^. Fourth, while prior DNA-launched flavivirus vaccines require electroporation for in vivo delivery^26,32,33^, we show for the first time that a PEI based reagent, in vivo-jetPEI, can be used as an alternative. According to the manufacturer’s protocol (Polyplus transfection), in vivo-jetPEI can be stored at the same temperature as DNA (-20 °C). Furthermore, once the DNA-reagent transfection mixture is prepared, it remains stable for up to 7 days when it is stored at 4 °C. By circumventing the need for an electroporator and by adopting a transfection reagent with simplified storage requirements, our approach maintains the convenience that DNA vaccines are meant to offer. Future work may focus on improving this reagent-based approach for vaccine delivery, as this would eventually make it easier to deploy DNA-launched VacDZ for mass vaccinations during outbreaks, especially in rural areas.

In this study, we demonstrate that VacDZ is a safe and effective vaccine candidate against ZIKV. VacDZ can be delivered as a live virus formulation or as a DNA-launched formulation. This convenience and flexibility would greatly benefit the future development and deployment of the vaccine, especially in vulnerable populations. All this makes VacDZ a promising vaccine candidate against ZIKV.

## Methods

### Cell culture and culture medium

This study utilised BHK-21 baby hamster kidney cells (ATCC® CCL-10™, USA), Huh-7 human hepatoma cells (kindly provided by Dr. Priscilla Yang, Harvard Medical School, USA), Vero African green monkey kidney cells (ATCC® CRL-1586™, USA), and C6/36 Aedes albopictus larvae cells (ATCC® CRL-1660™, USA). BHK-21 cells were cultured in Roswell Park Memorial Institute 1640 (RPMI) medium (Sigma-Aldrich) supplemented with 10% foetal calf serum (FCS). Vero and Huh-7 cells were cultured in Dulbecco’s Modified Eagle’s medium (DMEM) (Sigma-Aldrich) supplemented with 10% FCS. BHK-21, Vero, and Huh-7 cells were cultured in a 37 °C incubator with 5% CO_2_. C6/36 cells were cultured in Leibovitz-15 medium (L-15 medium) (Sigma-Aldrich) in a 28 °C incubator without CO_2_. All the RPMI medium and DMEM that were used in this study were also supplemented with 2 g/L of NaHCO_3_.

### Virus culture

This study utilised DENV2 strain 16681 (GenBank accession no. NC_001474.2), and ZIKV strain PRVABC59 (GenBank accession no. KU501215.1). This study also utilised VacDZ, a chimeric dengue/Zika virus, the construction of which is described below. DENV2-16681 was cultured in C6/36 cells with L-15 medium supplemented with 2% FCS. ZIKV and VacDZ were cultured in BHK-21 cells with RPMI supplemented with 2% FCS. Virus stocks were aliquoted and kept at -80 °C.

### Virus purification

Purified virus stock was produced for in vivo mouse studies. Supernatant from virus culture was first pre-clarified using a syringe driven 0.45-micron PES filter (Sartorius). The virus was then concentrated in Vivaspin Turbo 15 centrifugal concentrators, 100,000 MWCO (Sartorius). After concentration, buffer exchange was performed by washing the concentrated virus with phosphate-buffered saline (PBS) in the same centrifugal concentrator. The purified virus was then aliquoted and kept at -80 °C.

### Virus plaque assay

Titration of DENV2-16681, ZIKV, and VacDZ was done using plaque assay with BHK-21 cells. The day before inoculation, BHK-21 cells were seeded in a 24-well plate at a density of 5.0 × 10^4^ cells per well and left to grow overnight. On the day of inoculation, the virus stock was serially diluted 10-fold in RPMI medium supplemented with 2% FCS. The cell culture supernatant was removed from the BHK-21 cells, and 100 μl of the serially diluted virus stock was added to individual wells. Next, the virus was incubated with the cells in a 37 °C incubator with 5% CO_2_ for 1 h. After incubation, the diluted virus stock was removed and the BHK-21 cells were washed twice with 1 ml of PBS per well. After washing, the cells were overlaid with RPMI medium supplemented with 2% FCS, 2 g/L of NaHCO_3_, and 1% carboxymethyl cellulose (CMC) and then incubated in a 37 °C incubator with 5% CO_2_. Plaques were visualised by staining with a solution containing 10% paraformaldehyde and 1% crystal violet. For titration, incubation times for plaque assay were 6 days for DENV2-16681, 4 days for ZIKV, and 8 days for VacDZ. For comparison of plaque sizes of DENV2-16681 and VacDZ, incubation time was 6 days.

### Construction and production of VacDZ infectious clone plasmids

The infectious clone of the chimeric VacDZ, pVacDZ, was constructed using a combination of PCR, fusion PCR, and conventional molecular cloning techniques. The infectious clone was constructed by modifying our existing DENV2-16681 infectious clone, which uses the pSMART low copy plasmid (Lucigen) as a backbone^35^. To further stabilise our infectious clones, we had adopted the strategy of cloning an intron sequence into the DENV2-16681 infectious clone^37^. PCR primers were ordered from Integrated DNA Technologies. PCR and fusion PCR was performed using Q5 high-fidelity DNA polymerase (New England Biolabs). Restriction digestion was performed using restriction enzymes from New England Biolabs. DNA ligation was performed using T4 DNA ligase (New England Biolabs). Sanger sequencing was performed by First Base (Axil Scientific).

For construction of pVacDZ infectious clone, amplicons A to F were cloned by PCR using overlapping primers, or overlapping site mutagenesis primers (Supplementary Figure 1). Primers are shown in Supplementary Table 5. DENV2 sequences were cloned from our DENV2-16681 infectious clone, while ZIKV sequences were cloned from our ZIKV-PRVABC59 infectious clone. In particular, amplicon C consisted of the cDNA of the region encoding for the ZIKV prM signal sequence, prM protein, and Env protein. The overlapping primers were used to introduce overlapping sequences for fusion PCR. The site mutagenesis primers were used to introduce the attenuating mutations of VacDZ: 5′UTR-c57t, NS1-G53D and NS3-E250V. Amplicons A, B, and C were fused together by fusion PCR to form amplicon ABC. Amplicons D, E, and F were fused together to form amplicon DEF. Finally, amplicons ABC and DEF were fused together by fusion PCR to form amplicon ABCDEF. Amplicon ABCDEF was cloned into the DENV2-16681 infectious clone via NotI and XmaI restriction sites.

For construction of RDRP-defective mutant pVacDZ-ΔGDD, we used site mutagenesis primers to introduce an internal deletion of the ^662^GDD^664^ catalytic triad in the NS5 coding region. Primers are shown in Supplementary Table 5. Amplicons G and H were cloned by PCR using overlapping site mutagenesis primers (Supplementary Figure 2). The amplicons were fused together by fusion PCR to form amplicon GH. Amplicon GH was cloned into the pVacDZ infectious clone using the BsrGI and MluI restriction sites.

The infectious clone plasmids and pTet-Off Advanced plasmid were propagated in Stbl2 or Stbl3 *Escherichia coli* competent cells (Life Technologies, Thermo Fisher Scientific). The bacteria were cultured on Luria-Bertani (LB) agar plates or in LB medium at 28 °C. For the infectious clone plasmids, the medium was supplemented with 35 μg/ml of kanamycin. For the pTet-Off Advanced plasmid the medium was supplemented with 100 μg/ml of ampicillin. Plasmids were extracted and purified from bacteria using Qiagen Plasmid midi or maxi kits. For purification of in vivo grade plasmids for mouse studies, the plasmids were extracted and purified using Qiagen EndoFree Plasmid maxi kits.

### Rescue of VacDZ

Live virus was rescued from the pVacDZ infectious clone by DNA-launch^35,36^.

The day before transfection, BHK-21 cells were seeded in a 6-well plate at a density of 300,000 cells per well and left to grow overnight. On the day of inoculation, each well was transfected with a transfection mixture consisting of 1600 ng of pVacDZ plasmid, 400 ng of the pTet-Off Advanced accessory plasmid, 4 μl of jetPRIME transfection reagent (Polyplus transfection), and 200 μl of jetPRIME transfection buffer. The cells were then left overnight in a 37 °C incubator with 5% CO_2_. The day after transfection, the cell culture medium was changed to RPMI medium supplemented with 2% FCS. The cells were once again left in a 37 °C incubator with 5% CO_2_. The cells were observed daily and the supernatant containing the virus was harvested when the cell monolayer showed 30% or more cytopathic effect (CPE). Live VacDZ was then passaged in BHK-21 cells to produce the working virus stock.

### Growth kinetics and temperature sensitivity

The viral growth kinetic studies were performed for DENV2-16681, ZIKV, and VacDZ virus culture. Viral growth kinetics studies were performed in BHK-21, Vero, Huh-7, and C6/36 cells. Virus culture and virus dilution were performed with RPMI medium supplemented with 2% FCS for BHK-21 cells, DMEM supplemented with 2% FCS for Vero and Huh-7 cells, and L-15 medium supplemented with 2% FCS for C6/36 cells.

The day before inoculation, cells were seeded in 6-well plates and left to grow overnight. Seeding densities per well are as follows: 2.1 × 10^5^ cells for BHK-21 cells, 3.3 × 10^5^ cells for Vero cells, 3 × 10^5^ cells for Huh-7 cells, and 3 × 10^5^ cells for C6/36 cells. On the day of inoculation, the virus stocks for DENV2-16681, ZIKV, or VacDZ were diluted to a multiplicity of infection (MOI) of 1.0. The cell culture supernatant was removed from the 6-well plates, and 1 ml of the diluted virus was added to the wells. The cells were then incubated in a 37 °C incubator with 5% CO_2_ for 1 h. The diluted virus was removed and the wells were topped up with 4 ml of the appropriate virus culture medium. BHK-21, Vero, and Huh-7 cells were incubated in a 37 °C incubator with 5% CO_2_. C6/36 cells were incubated in a 28 °C incubator. To perform temperature sensitivity studies for DENV2-16681, ZIKV, and VacDZ in BHK-21 cells, the same process was repeated with an extra set of plates that were kept in a 39 °C incubator with 5% CO_2_. Every day, starting with the day after inoculation, virus supernatant was harvested from three wells and stored at -80 °C. The viruses were then titrated using plaque assay.

### Immunofluorescence assay

Immunofluorescence assay was performed in BHK-21 cells that were plated on glass slides. The cells were infected with DENV2-16681, ZIKV or VacDZ and then incubated in a 37 °C incubator with 5% CO_2_. Two days after infection, the cells were fixed and permeabilised with methanol at -20 °C. For immunofluorescence staining, the primary antibodies were mouse monoclonal anti-DENV/ZIKV NS1 protein (DN2, Abcam) at a dilution of 1:10, and rabbit monoclonal anti-ZIKV envelope protein (Ab00812-23.0, Absolute antibody) at a dilution of 1:200. Although DN2 is described as an anti-DENV NS1 protein antibody, we have found that it is cross reactive with ZIKV as well (Supplementary Figure 3). Secondary antibodies were FITC-conjugated goat anti-rabbit IgG (H + L) (F-2765, Thermo Fisher Scientific) at a dilution of 1:500 and Alexa 594-conjugated goat anti-mouse IgG (H + L) (A-11005, Thermo Fisher Scientific) at a dilution of 1:500. The slides were then mounted on microscope slides using Fluoroshield with DAPI (Sigma-Aldrich). Images were taken with an Olympus IX81 fluorescence microscope equipped with a UPlanApo 100x microscope objective lens (numerical aperture 1.35, Olympus) and Photometrics CoolSnap HQ CCD camera. Image acquisition was performed with MetaMorph software for Olympus.

### Next generation sequencing of VacDZ

Viral RNA was extracted using acid phenol:chloroform (Life Technologies, Thermo Fisher Scientific). Viral cDNA synthesis was performed using Maxima H Minus reverse transcriptase using virus specific primers (Thermo Fisher Scientific). The reversion rates of the three attenuating mutations of VacDZ were analysed by next-generation sequencing (NGS). Library preparation, NGS, and data analysis services were provided by Omics Drive Pte Ltd. Library preparation was performed with VAHTS Universal DNA Library Prep Kit for Illumina. NGS was performed on the Novaseq 150PE system. Fastq sequences were aligned to the reference genome using BWA to generate the bam file. The bam file was sorted filtered and sorted with SAMTOOLS. Reversions were detected using GATK.

### Ethical approval

All animal experiments were reviewed and approved by the National University of Singapore Institutional Animal Care and Use Committee (IACUC) under protocol number R18-0488.

### Neurovirulence studies in suckling mice

Neurovirulence studies were conducted in newborn outbred white ICR mice (InVivos Pte Ltd). The newborn mice were inoculated within 24 h of birth with either DENV2-16681, ZIKV, or VacDZ or with a PBS vehicle control. The newborn mice were inoculated intracranially with 1 μl of PBS or virus at a dose of 1, 10, or 100 PFU per mouse. The mice were observed daily for clinical signs such as weight loss, wobbling gait, limb weakness or dragging, paralysis, hunching, laboured breathing, or inactivity, and killed when they reached a humane endpoint. The clinical scoring methodology that we used is shown in Supplementary Table 6. Four weeks after inoculation, the surviving mice were killed.

### Pathogenicity studies in AG129 mice

Pathogenicity studies were conducted in 5–8 weeks old AG129 mice (InVivos Pte Ltd). AG129 mice were inoculated with either ZIKV, or VacDZ or a PBS vehicle control. The mice were inoculated intraperitoneally with 100 μl of virus or PBS vehicle control. For ZIKV, the dose was 1000, or 10,000 PFU per mouse. For VacDZ the dose was 1 × 10^6^ PFU per mouse. The mice were then observed daily for clinical signs such as weight loss, limb weakness or dragging, paralysis, hunching, laboured breathing, inactivity, or ruffled fur and killed when they reached a humane endpoint. The clinical scoring methodology that we used is shown in Supplementary Table 6. Four weeks after inoculation the surviving mice were killed.

### Immunogenicity studies in AG129 mice

Immunogenicity studies were conducted in 5–8 weeks old AG129 mice (InVivos Pte Ltd).

For immunogenicity studies with live VacDZ, the dose was 10,000 PFU per mouse, in a volume of 100 μl. A total of 100 μl of PBS was used as a vehicle control. For immunogenicity studies with DNA-launched VacDZ, the dose was a transfection mixture consisting of 80 μg of pVacDZ and 20 μg of pTet-Off Advanced, 16 µL in vivo-jetPEI (Polyplus transfection), in 500 µl of a 5% glucose solution. In total 500 µl of a 5% glucose solution was used as a vehicle control. The replication defective control was a transfection mixture consisting of 80 μg of pVacDZ-ΔGDD mutant and 20 μg of pTet-Off Advanced, 16 µL in vivo-jetPEI (Polyplus transfection), in 500 µl of a 5% glucose solution.

The mice were first vaccinated via intraperitoneal injection with live VacDZ, DNA-launched VacDZ, or their respective controls. Four weeks after the initial vaccination the mice were boosted with the same dose of their respective vaccine or control. Four weeks after boosting (8 weeks after initial vaccination), the mice were killed and their organs and blood were harvested for analysis.

### Protective immunity studies in AG129 mice

Protective immunity studies were conducted in 5–8 weeks old AG129 mice (InVivos Pte Ltd).

For protective immunity studies with live VacDZ, the dose was 10,000 PFU per mouse, in a volume of 100 μl. In total 100 μl of PBS was used as a vehicle control. For protective immunity studies with DNA-launched VacDZ, the dose was a transfection mixture consisting of 80 μg of pVacDZ and 20 μg of pTet-Off Advanced, 16 µL in vivo-jetPEI (Polyplus transfection), in 500 µl of a 5% glucose solution. 500 µl of a 5% glucose solution was used as a vehicle control. The replication defective control was a transfection mixture consisting of 80 μg of pVacDZ-ΔGDD mutant and 20 μg of pTet-Off Advanced, 16 µL in vivo-jetPEI (Polyplus transfection), in 500 µl of a 5% glucose solution.

For the protective immunity studies, mice were vaccinated with a single dose of vaccine or control, and then challenged with ZIKV. The mice were first vaccinated via intraperitoneal injection with live VacDZ, DNA-launched VacDZ, or their respective controls. Four weeks after the initial vaccination the mice were challenged with a lethal dose of 10^5^ PFU of ZIKV (volume of 100 μl) via intraperitoneal inoculation. The mice were then observed daily for clinical signs such as weight loss, limb weakness or dragging, paralysis, hunching, laboured breathing, inactivity, or ruffled fur and killed when they reached a humane endpoint. The clinical scoring methodology that we used is shown in Supplementary Table 6. Four weeks after lethal challenge with ZIKV (eight weeks after initial vaccination) the surviving mice were killed, and their organs and blood were harvested for analysis.

### Plaque reduction neutralisation test

Plaque reduction neutralisation test (PRNT) was performed in BHK-21 cells. The day before inoculation, BHK-21 cells were seeded in a 24-well plate and left to grow overnight. On the day of inoculation, the ZIKV stock was diluted to a concentration of 50 PFU per 50 μl in RPMI medium supplemented with 2% FCS. Mouse serum was first heat-inactivated at 55 °C for 30 min. The mouse serum was then diluted 10-fold to give a starting dilution of 1:10. The serum was then further serially diluted two-fold to give dilutions ranging from 1:20 up to 1:20480. The serially diluted mouse serum was then mixed with an equal volume of the diluted ZIKV and incubated at 37 °C for 1 h.

After incubation the BHK-21 cells were inoculated with the ZIKV-serum mixtures (volume of 100 μl per well). Next, the virus was incubated with the cells in a 37 °C incubator with 5% CO_2_ for 1 h. After incubation, the virus was removed and the BHK-21 cells were washed twice with 1 ml of PBS per well. After washing, the cells were overlaid with RPMI medium supplemented with 2% FCS, 2 g/L of NaHCO_3_, and 1% carboxymethyl cellulose (CMC) and then incubated in a 37 °C incubator with 5% CO_2_.

The neutralising antibody titre is defined as the highest serum dilution that reduced the ZIKV plaque count by at least 50% in three technical replicates, and mice which had a neutralising antibody titre of 1:10 or more (≥ 1:10) were considered to have seroconverted^11^. For statistical analysis, mice that did not seroconvert were considered to have a neutralisation titre equal to the detection limit of our PRNT assay (1:10), giving a log_10_(titre) = 1.

### ELIspot assay

ELIspot assay was conducted using mouse T-cell ELIspot kits from Immunospot in the precoated 96-well strip format to determine the type 1 or type 2 T helper cell (Th1 or Th2) response. Interferon γ (IFNγ) ELIspot assay was performed to determine the Th1 response and interleukin 4 ELIspot assay was performed to determine the Th2 response.

To activate ZIKV-specific T-cells, we used an activation mixture consisting of 66 μl of CTL media (Immunospot) and 2.5 × 10^5^ PFU of ZIKV that was resuspended in 34 μl of RPMI medium supplemented with 2% FCS. The control mixture consisted of 66 μl of CTL media and 34 μl of RPMI medium supplemented with 2% FCS.

On the day that the mouse spleens were harvested, the ELIspot plates, the activation mixture, and the control mixture were prepared prior to splenocyte isolation. First, the plates were washed once with PBS, and then the PBS was removed. Next, the activation mixture was added to half the wells (100 μl per well), and the control mixture was added to the remaining wells (100 μl per well).

To prepare mouse splenocytes for ELIspot assay, freshly harvested mouse spleens were disassociated using a Greiner 70 µM easy strainer and resuspended in serum free RPMI medium. The isolated splenocytes were counted, and then spun down and resuspended in CTL media (Immunospot) at a density of 5 × 10^6^ cells/ml. Next, the resuspended splenocytes were added to the wells with the activation mixture or control mixture at a density of 5 × 10^5^ cells per well (100 μl of the resuspended cells). The plates were then incubated for 24 h in a 37 °C incubator with 5% CO_2_. After the incubation, the plates were washed and ELIspot assay was performed according to the manufacturer’s instructions.

### Statistical analysis

Statistical analysis was performed using one way Anova (Excel, Microsoft), and post-hoc analysis was performed using Tukey HSD (https://astatsa.com/OneWay_Anova_with_TukeyHSD/; accessed June 2020).

### Reporting summary

Further information on experimental design is available in the Nature Research Reporting Summary linked to this paper.

## Supplementary information

Supplementary Information

Reporting Summary

## Data Availability

All the relevant data is presented in this paper or within the supplementary materials.

## Supplementary information

**Supplementary information** The online version contains supplementary material available at 10.1038/s41541-021-00282-y.
