# Supplementary material for: A single-dose live attenuated chimeric vaccine candidate against Zika virus
Source: NPJ Vaccines. 2021 Jan 29;6:20. doi: 10.1038/s41541-021-00282-y (PMC7846741; doi:10.1038/s41541-021-00282-y)
Supplement: Supplementary file 1 — Supplementary Information [file 41541_2021_282_MOESM1_ESM.pdf]

Supplementary Figures

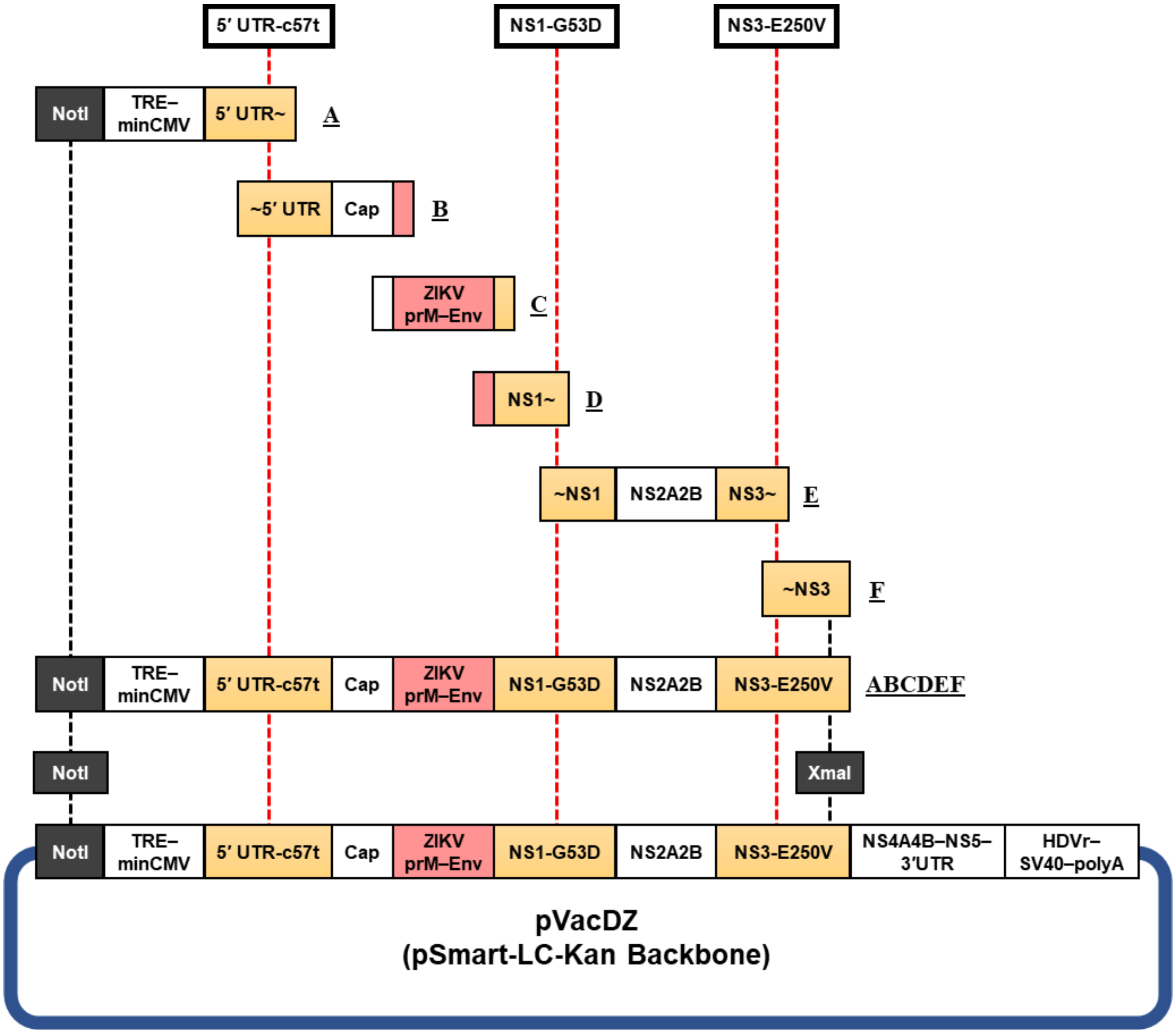

Supplementary Figure 1. Construction of chimeric dengue/Zika virus VacDZ infectious clone. The infectious clone of VacDZ, called pVacDZ, was constructed using PCR, fusion PCR, and conventional molecular cloning techniques. A, B, C, D, E, and F indicate PCR amplicons that were amplified using primers, fusion PCR primers, or site-mutagenesis primers (**Table S4**). The PCR amplicons were fused by fusion PCR to ultimately form amplicon ABCDEF, which was cloned into a DENV2 infectious clone using the NotI and XmaI restriction digestion sites.

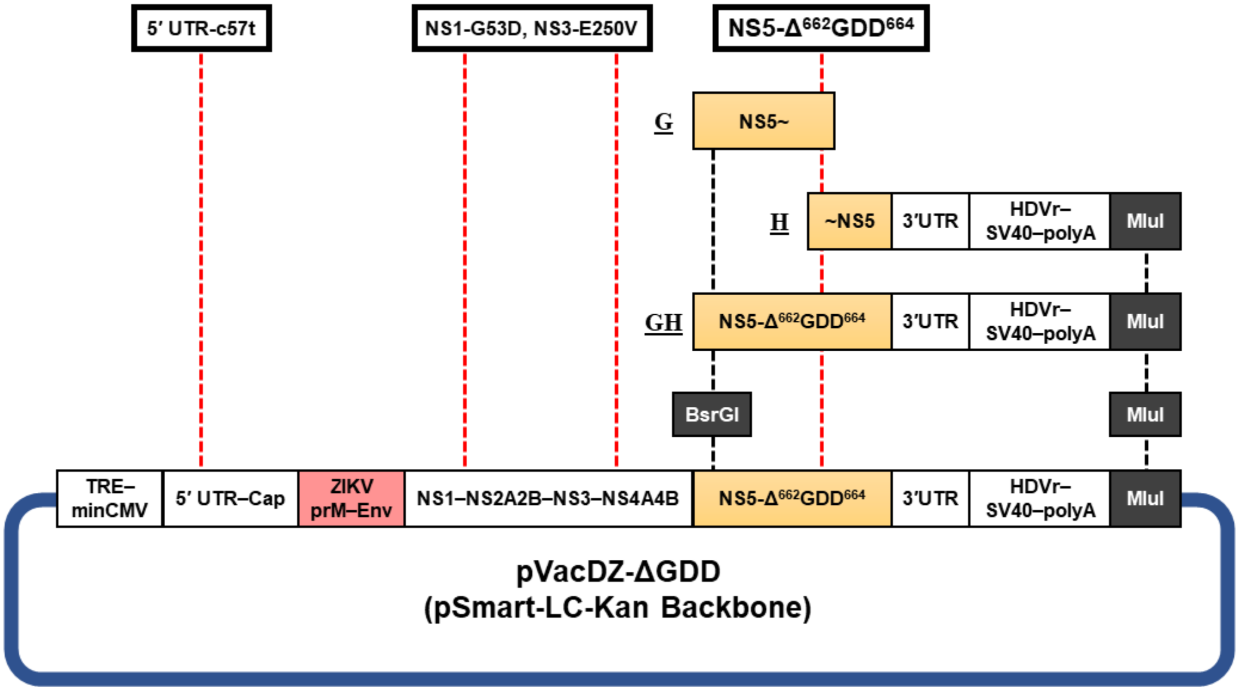

Supplementary Figure 2. Construction of NS5 ΔGDD mutant of VacDZ. The infectious clone pVacDZ-ΔGDD with a deletion of the GDD catalytic triad of the NS5 protein RNA-dependent RNA polymerase was constructed using PCR, fusion PCR, and conventional molecular cloning techniques. G and H indicate PCR amplicons that were amplified using site-mutagenesis primers. The PCR amplicons were fused by fusion PCR to form amplicon GH, which was cloned into a pVacDZ infectious clone using the BsrGI and MluI restriction digestion sites.

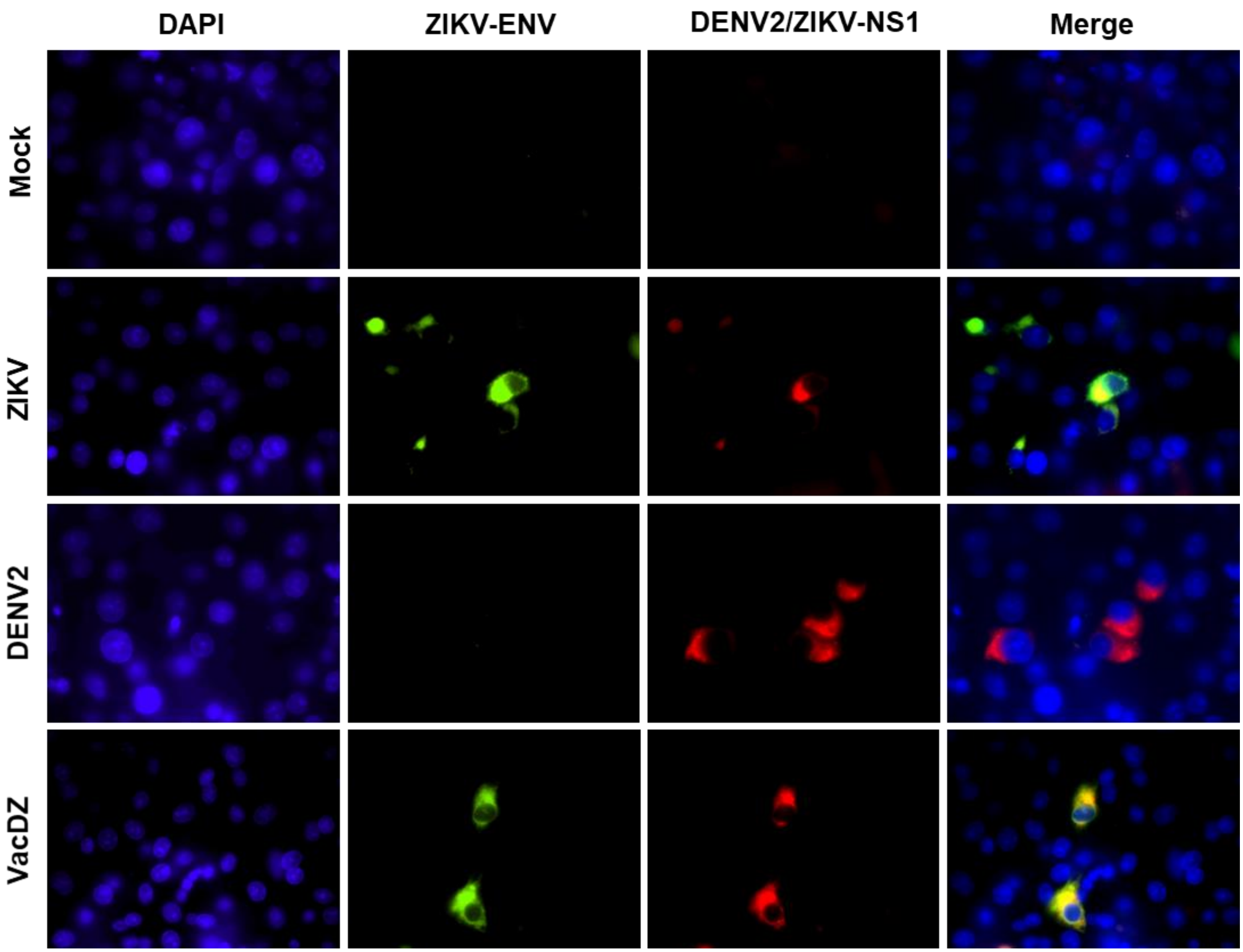

Supplementary Figure 3. Immunofluorescence assay for ZIKV-Env expression. BHK-21 cells were infected with ZIKV, DENV2-16681, or VacDZ. The cells were fixed and immunofluorescence assay was used to analyse expression of DENV2 or ZIKV NS1 protein (red), or ZIKV envelope protein (green). Host nuclei were stained with DAPI (blue).

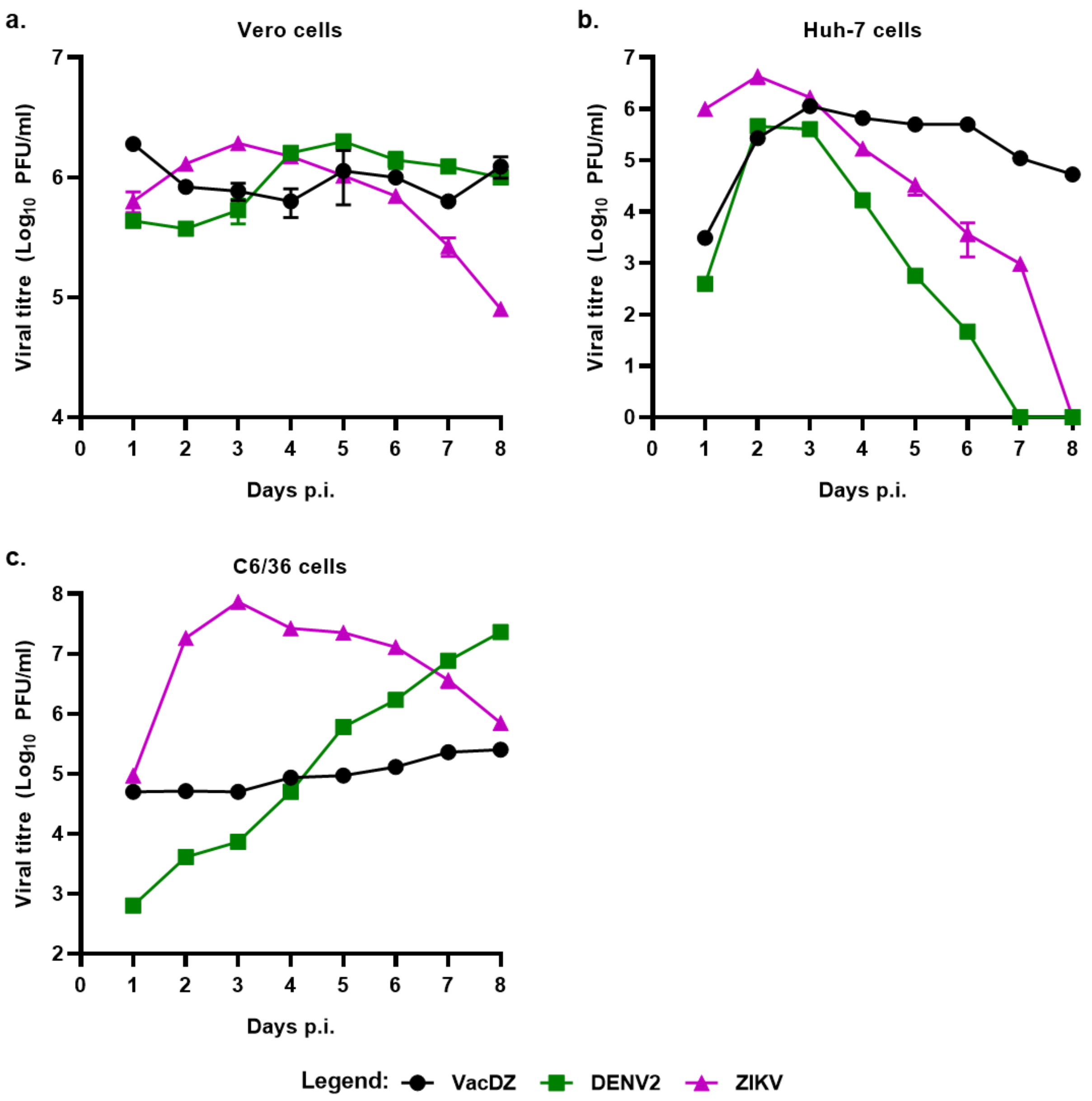

Supplementary Figure 4. Virus replication kinetics. Viral replication kinetics for VacDZ, DENV2-16681 and ZIKV in different cell lines. The indicated cell lines were infected with the different viruses at an MOI of 1 and the viral titres were analysed by using plaque assay. **(a)** Vero African green monkey kidney cells. **(b)** Huh-7 human hepatoma cells. **(c)** C6/36 *Aedes albopictus* mosquito cells. Data represents the mean of three replicates.

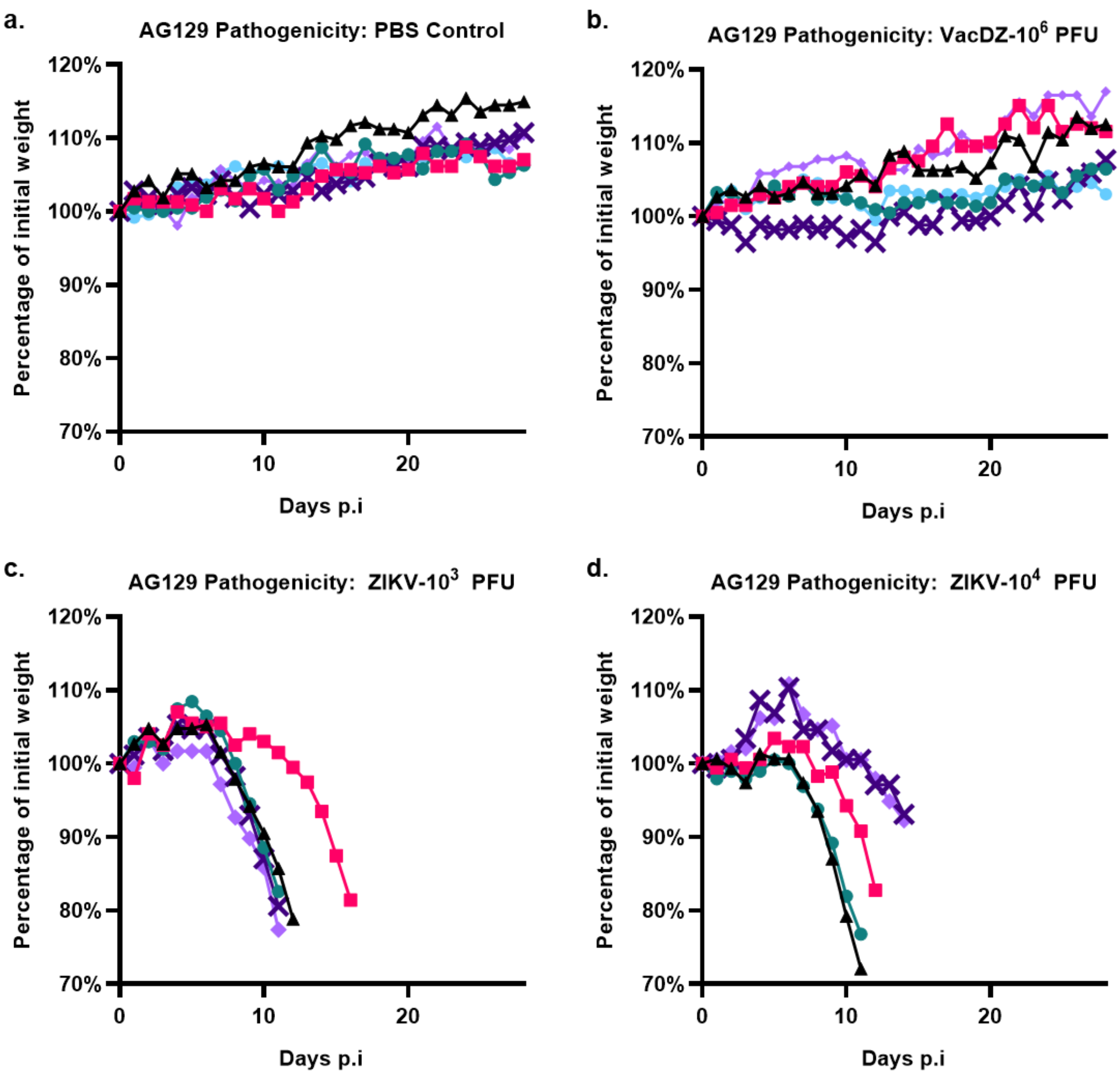

Supplementary Figure 5. AG129 mouse weights during pathogenicity studies. 5-8 weeks old AG129 mice were challenged with the indicated doses of VacDZ, ZIKV or PBS control via intraperitoneal inoculation. The mice were kept for four weeks and observed daily for clinical symptoms and euthanised when they reached a humane endpoint. Each line represents one mouse. **(a)** PBS vehicle control group, n=6. **(b)** Live VacDZ, 10^6 PFU per mouse, n=6. **(c)** ZIKV, 10^3 PFU per mouse, n=5. **(d)** ZIKV, 10^4 PFU per mouse, n=5.

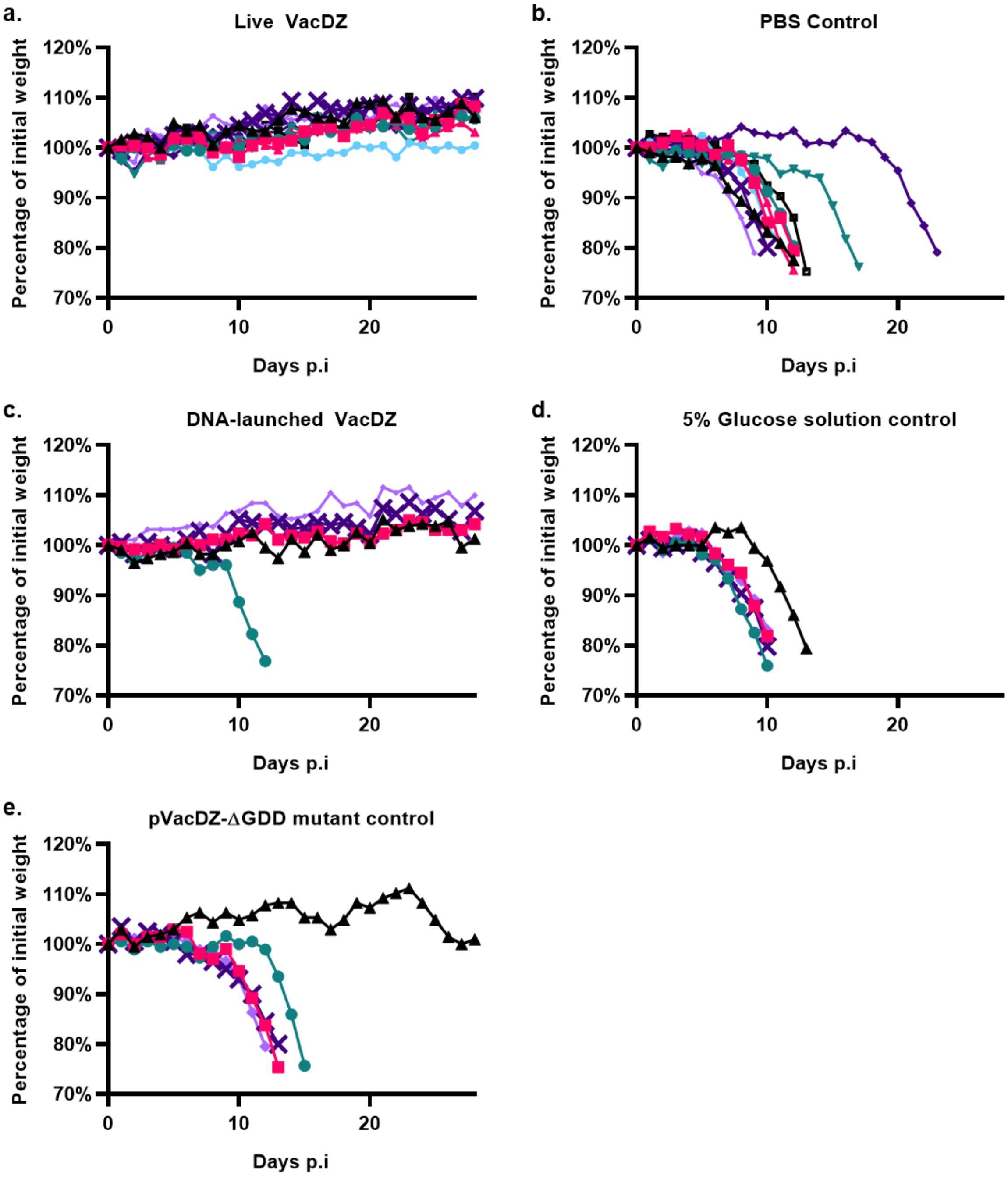

Supplementary Figure 6. Mouse weights for protective immunity studies in AG129 mice. 5-8 weeks old AG129 mice were inoculated intraperitoneally with the indicated vaccine or control. Four weeks after vaccination they were challenged with a lethal dose of 10^5 PFU of ZIKV per

mouse. The mice were kept for four weeks and observed daily for clinical symptoms and euthanised when they reached a humane endpoint. Each line represents one mouse. Vaccination doses: **(a)** VacDZ (10,000 PFU per mouse, n=11). **(b)** PBS vehicle control (n=10). **(c)** DNA-launched VacDZ (80 μg of pVacDZ and 20 μg of pTet-Off Advanced per mouse, n=5). **(d)** 5% glucose solution vehicle control (n=5). **(e)** pVacDZ-ΔGDD replication defective mutant (80 μg of pVacDZ-ΔGDD and 20 μg of pTet-Off Advanced per mouse, n=5).

Supplementary Tables

Attenuation Loci	Nucleotide Position	VacDZ Nucleotide	Wildtype Nucleotide	Change	Reversion Rate
DV2-5′UTR	5′UTR-57	T	C	T -> C	<1%
DV2-NS1	NS1-158	A	G	A -> G	<1%
DV2-NS3	NS3-749	T	A	T -> A	<1%

Supplementary Table 1. Reversion rate of key attenuating mutations of VacDZ. VacDZ was serial passaged in BHK-21 cells. At passage 10 viral RNA was extracted and the reversion rates of the attenuating mutations were analysed by using next-generation sequencing.

Virus	Dose (PFU per mouse)	Number of mice Surviving/Total	Mortality Rate
VacDZ	1	11/11	0%
VacDZ	10	11/11	0%
VacDZ	100	11/11	0%
DENV2-16681	10	4/12	66.7%
DENV2-16681	100	3/12	75%
ZIKV	1	2/10	80%
ZIKV	10	0/7	100%
ZIKV	100	0/14	100%
PBS control	NA	6/6	0%

Supplementary Table 2. Details of neurovirulence study in suckling mice. Newborn mice were challenged with the indicated doses of VacDZ, DENV2-16681, or ZIKV via intracranial inoculation.

Vaccination Agent	No. of Mice	Seroconversion Rate	PRNT Titre Geometric Mean
Live VacDZ	8	100%	1:3044.37
PBS control	6	0%	ND
DNA-launched VacDZ	5	100%	1:1114.3
5% Glucose solution control	5	0%	ND
pVacDZ-ΔGDD control	4	0%	ND

Supplementary Table 3. Seroconversion and neutralisation titres of vaccinated mice. AG129 mice were vaccinated and boosted four weeks apart with live VacDZ, DNA-launched VacDZ or their respective controls. Four weeks after boosting, mouse serum was harvested and induction of neutralising antibodies against ZIKV was analysed using PRNT. ND: non-detected, titres were below the detection limit of the PRNT.

Vaccination Agent	No. of Mice	Survival Rate	Seroconversion Rate (surviving mice)	PRNT Titre Geometric Mean
Live VacDZ	11	100%	100%	1:3044.37
PBS control	10	0%	NA	NA
DNA-launched VacDZ	5	80%	100%	1:1114.3
pVacDZ-ΔGDD control	5	20%	100%	1:10240
5% Glucose control	5	0%	NA	NA

Supplementary Table 4. Survival rate, seroconversion and neutralisation titres of vaccinated mice. AG129 mice were vaccinated with live VacDZ, DNA-launched VacDZ or their respective controls and then challenged four weeks later with a lethal dose of ZIKV (10^5 PFU per mouse). Four weeks after lethal challenge, mouse serum was harvested from the surviving mice and induction of neutralising antibodies against ZIKV was analysed by using plaque reduction neutralisation test. NA: not applicable, there were no surviving mice to perform PRNT.

Amplicon	Corresponding region	PCR primers
A	NotI site–TRE–CMVmin–5′UTR~	5′: CAGTCCAGTTACGCTGGAGTC<br>3′: GTTAGAACTACATTGAGCTTAGCTCCCTCAAAG
B	~5′UTR–Cap–prM signal sequence	5′: GCTAAGCTCAATGTAGTTCTAACAGTTTTTTAATTAG<br>3′: CGACACTAGTGCCTGCAGATCTGCGTCTC
C	ZIKV: prM signal sequence–prM–Env	5′: GATCTGCAGGCACTAGTGTCGGAATTGTTGGC<br>3′: GCAACCACTATCAGCAGAGACGGCTGTGGA
D	NS1~	5′: GTCTCTGCTGATAGTGGTTGCGTTGTGAGCT<br>3′: TCCACAAATGtCCTCTTCATGGGCTTTCTG
E	~NS1–NS2A–NS3~	5′: CATGAAGAGGaCATTTGTGGAATCCGCTCA<br>3′: CCCGGTGTGCACAGCTCTGATGGCTGGGGTC
F	~NS3	5′: ATCAGAGCTGTGCACACCGGGCGGGAGATTGT<br>3′: CTTTCTTCCGGCTGCAAATTCC
ABC	NotI–TRE–CMVmin–5′UTR–NS1~	5′: CAGTCCAGTTACGCTGGAGTC<br>3′: GCAACCACTATCAGCAGAGACGGCTGTGGA
DEF	~NS1–NS3	5′: GTCTCTGCTGATAGTGGTTGCGTTGTGAGCT<br>3′: CTTTCTTCCGGCTGCAAATTCC
ABCDEF	NotI–TRE–CMVmin–5′UTR–NS1–NS3	5′: CAGTCCAGTTACGCTGGAGTC<br>3′: CTTTCTTCCGGCTGCAAATTCC
G	NS5~	5′: GGAACTGGCAACATAGGAGAGA<br>3′: GTTTCACAACACAACTGATGGCCATTCTTGATAACC
H	~NS5–3′UTR–HDVr–SV40–polyA–MluI site	5′: GGCCATCAGTTGTGTTGTGAAACCTTTAGATGAC<br>3′: GGTCAGGTATGATTTAAATGGTCAGT
GH	NS5–3′UTR–HDVr–SV40–polyA–MluI site	5′: GGAACTGGCAACATAGGAGAGA<br>3′: GGTCAGGTATGATTTAAATGGTCAGT

Supplementary Table 5. Primer sequences for PCR. NS1~, ~NS1, ~NS3… etc indicates partial fragment of the indicated coding region.

Score	1	2	3
Activity	1a<br>Abnormal posture (e.g. hunched), lethargy.	2a<br>Inactive, overactive, huddled.	3a<br>Moribund, seizure (no movement, uncontrollable behaviour).
Movement	1b<br>Slight incoordination, (Appears to limp while walking, wobbling but doesn’t really drop).	2b<br>1 limb paralysis, slight tremor, unsteady gait (wobbling and falling while trying to walk), severe limp, lower pelvis close to ground, feet pointing away from body.	3b<br>2 limb paralysis, severe tremor, severe ataxia, cannot roll over, difficulty moving forward, dragging abdomen on ground.
Breathing	1c<br>Rapid shallow.	2c<br>Rapid abdominal.	3c<br>Laboured, blue.
Body Weight	1d<br><br>5% decrease over 24h	2d<br>10% decrease over 24h, or cumulative weight loss >15%.	3d<br>Weight loss >10% over 24h, or cumulative weight loss >20%.

Supplementary Table 6. Clinical scoring methodology. For mouse pups, the humane endpoint is reached when the pup reaches a cumulative score of 3 (e.g activity, movement, and breathing scores of 1 + 1 + 1). For adult mice, humane endpoint is reached when any individual category reaches a score of 3 (e.g activity = 3, or movement = 3), or when the mouse reaches a cumulative score of 6.
