# Supplementary material for: A single-dose live attenuated chimeric vaccine candidate against Zika virus
Source: NPJ Vaccines. 2021 Jan 29;6:20. doi: 10.1038/s41541-021-00282-y (PMC7846741; doi:10.1038/s41541-021-00282-y)
Supplement: Supplementary file 2 — Reporting Summary [file 41541_2021_282_MOESM2_ESM.pdf]

nature research

Corresponding author(s): Justin Jang Hann Chu

Last updated by author(s): Nov 29, 2020

Reporting Summary

Nature Research wishes to improve the reproducibility of the work that we publish. This form provides structure for consistency and transparency in reporting. For further information on Nature Research policies, see our Editorial Policies and the Editorial Policy Checklist.

Statistics

For all statistical analyses, confirm that the following items are present in the figure legend, table legend, main text, or Methods section.

n/a	Confirmed	
☐	☒	The exact sample size (n) for each experimental group/condition, given as a discrete number and unit of measurement
☐	☒	A statement on whether measurements were taken from distinct samples or whether the same sample was measured repeatedly
☐	☒	The statistical test(s) used AND whether they are one- or two-sided<br>*Only common tests should be described solely by name; describe more complex techniques in the Methods section.*
☐	☒	A description of all covariates tested
☒	☐	A description of any assumptions or corrections, such as tests of normality and adjustment for multiple comparisons
☐	☒	A full description of the statistical parameters including central tendency (e.g. means) or other basic estimates (e.g. regression coefficient) AND variation (e.g. standard deviation) or associated estimates of uncertainty (e.g. confidence intervals)
☒	☐	For null hypothesis testing, the test statistic (e.g. F, t, r) with confidence intervals, effect sizes, degrees of freedom and P value noted<br>*Give P values as exact values whenever suitable.*
☒	☐	For Bayesian analysis, information on the choice of priors and Markov chain Monte Carlo settings
☒	☐	For hierarchical and complex designs, identification of the appropriate level for tests and full reporting of outcomes
☒	☐	Estimates of effect sizes (e.g. Cohen's d, Pearson's r), indicating how they were calculated

Our web collection on statistics for biologists contains articles on many of the points above.

Software and code

Policy information about availability of computer code

Data collection	No software used.
Data analysis	Statistical analysis was performed using one way Anova (Excel, Microsoft), and post-hoc analysis was performed using Tukey HSD (https://astatsa.com/OneWay_Anova_with_TukeyHSD/; accessed June 2020).

For manuscripts utilizing custom algorithms or software that are central to the research but not yet described in published literature, software must be made available to editors and reviewers. We strongly encourage code deposition in a community repository (e.g. GitHub). See the Nature Research guidelines for submitting code & software for further information.

Data

Policy information about availability of data

All manuscripts must include a data availability statement. This statement should provide the following information, where applicable:

- Accession codes, unique identifiers, or web links for publicly available datasets
- A list of figures that have associated raw data
- A description of any restrictions on data availability

All the relevant data is presented in this paper or within the supplementary materials.

Field-specific reporting

Please select the one below that is the best fit for your research. If you are not sure, read the appropriate sections before making your selection.

☒ Life sciences ☐ Behavioural & social sciences ☐ Ecological, evolutionary & environmental sciences

For a reference copy of the document with all sections, see nature.com/documents/nr-reporting-summary-flat.pdf

Life sciences study design

All studies must disclose on these points even when the disclosure is negative.

Sample size	Sample sizes for animal studies were determined based on prior studies, and were approved by NUS Institutional Animal Care and Use Committee.
Data exclusions	No data excluded.
Replication	In vitro experiments were verified in three independent experiments.<br>Treatment group sizes for mouse studies are indicated in the manuscript.
Randomization	Mice that were housed to together were assigned to the same treatment group. For suckling mice, each litter was assigned to one treatment group. For adult mice, if 4 or 5 mice were housed together, they were split into groups of 2 or 3 mice that were housed together, and each of those groups were assigned to different treatment groups.
Blinding	Blinding was not possible for animal work as the number of investigators involved in animal work was too small.

Reporting for specific materials, systems and methods

We require information from authors about some types of materials, experimental systems and methods used in many studies. Here, indicate whether each material, system or method listed is relevant to your study. If you are not sure if a list item applies to your research, read the appropriate section before selecting a response.

Materials & experimental systems

n/a	Involved in the study	
☐	☒	Antibodies
☐	☒	Eukaryotic cell lines
☒	☐	Palaeontology and archaeology
☐	☒	Animals and other organisms
☒	☐	Human research participants
☒	☐	Clinical data
☒	☐	Dual use research of concern

Methods

n/a	Involved in the study	
☒	☐	ChIP-seq
☒	☐	Flow cytometry
☒	☐	MRI-based neuroimaging

Antibodies

Antibodies used	Primary antibodies were mouse monoclonal anti-dengue virus and Zika virus NS1 protein (DN2, Abcam), and rabbit monoclonal anti-ZIKV envelope protein (Ab00812-23.0, Absolute antibody). Secondary antibodies were FITC-conjugated goat anti-rabbit IgG (H+L) (F-2765, Thermo Fisher Scientific) and Alexa 594-conjugated goat anti-mouse IgG (H+L) (A-11005, Thermo Fisher Scientific).
Validation	The validation of the primary antibodies is based upon validation done by the supplier, as well as validation using the positive controls, as described in the manuscript.

Eukaryotic cell lines

Policy information about cell lines

Cell line source(s)	This study utilised BHK-21 baby hamster kidney cells (ATCC® CCL-10™, USA), Huh-7 human hepatoma cells (kindly provided by Dr. Priscilla Yang, Harvard Medical School, USA), Vero African green monkey kidney cells (ATCC® CRL-1586™, USA), and C6/36 Aedes albopictus larvae cells (ATCC® CRL-1660™, USA).
Authentication	Cell lines were not authenticated.
Mycoplasma contamination	Cell lines tested negative for mycoplasma.

Commonly misidentified lines (See ICLAC register)	None.

Animals and other organisms

Policy information about studies involving animals; ARRIVE guidelines recommended for reporting animal research

Laboratory animals	Newborn outbred white ICR mice, male and female, less than 24 hours old. Adult AG129 mice, male and female, 5-8 weeks old.
Wild animals	Study did not involve wild animals.
Field-collected samples	Study did not involve samples collected in the field.
Ethics oversight	NUS Institutional Animal Care and Use Committee (IACUC)

Note that full information on the approval of the study protocol must also be provided in the manuscript.
